# Use of complementary nucleobase-containing synthetic polymers to prepare complex self-assembled morphologies in water†
†Electronic supplementary information (ESI) available: Characterization of monomers, polymers and particles: NMR, SEC, TEM, SAXS, and DLS. See DOI: 10.1039/c6py00263c



**DOI:** 10.1039/c6py00263c

**Published:** 2016-04-06

**Authors:** Yan Kang, Anaïs Pitto-Barry, Marianne S. Rolph, Zan Hua, Ian Hands-Portman, Nigel Kirby, Rachel K. O’Reilly

**Affiliations:** a Department of Chemistry , University of Warwick , Gibbet Hill Road , Coventry , CV4 7AL , UK . Email: Rachel.OReilly@warwick.ac.uk; b School of Life Sciences , University of Warwick , Gibbet Hill Road , Coventry , CV4 7AL , UK; c Australian Synchrotron , 800 Blackburn Road , Clayton Vic 3168 , Australia

## Abstract

Amphiphilic nucleobase-containing block copolymers with poly(oligo(ethylene glycol) methyl ether methacrylate) as the hydrophilic block and nucleobase-containing blocks as the hydrophobic segments were successfully synthesized using RAFT polymerization and then self-assembled *via* solvent switch in aqueous solutions. Effects of the common solvent on the resultant morphologies of the adenine (A) and thymine (T) homopolymers, and A/T copolymer blocks and blends were investigated. These studies highlighted that depending on the identity of the common solvent, DMF or DMSO, spherical micelles or bicontinuous micelles were obtained. We propose that this is due to the presence of A–T interactions playing a key role in the morphology and stability of the resultant nanoparticles, which resulted in a distinct system compared to individual adenine or thymine polymers. Finally, the effects of annealing on the self-assemblies were explored. It was found that annealing could lead to better-defined spherical micelles and induce a morphology transition from bicontinuous micelles to onion-like vesicles, which was considered to occur due to a structural rearrangement of complementary nucleobase interactions resulting from the annealing process.

## Abstract 2

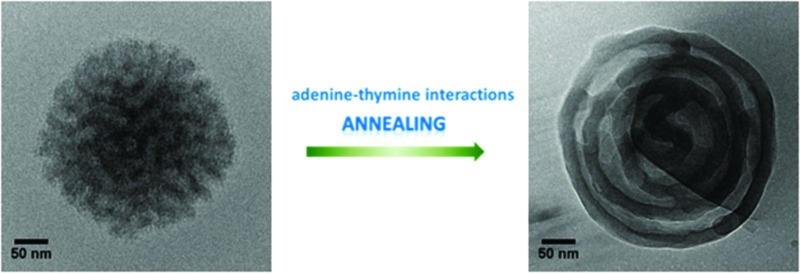
Amphiphilic block copolymers with synthetic nucleobase-containing blocks as the hydrophobic segments were successfully synthesized using RAFT polymerisation and then self-assembled *via* solvent switch in aqueous solutions.

## Introduction

The incorporation of nucleobase functionalities in synthetic polymer chemistry is of interest, as these polymers can be applied in various fields such as templated polymerizations^1–4^ and supramolecular self-assemblies.^5–10^ For example, giant vesicles,^5^ Au-containing particles,^11^ and thermally reversible microspheres^8^ were demonstrated to form based on the complementary interactions between thymine and diaminopyridine functionalities among polymers. Rods were observed to form through the self-assembly of adenine-containing norbornene block copolymers, a morphology that was unexpected given the large corona : core ratio (DP of corona : DP of core = 50 : 5).^7^ In our previous work, the preparation of a range of nucleobase-containing morphologies by reversible addition–fragmentation chain transfer (RAFT) dispersion polymerization was demonstrated.^10,12^ However, these self-assemblies were mostly prepared in organic solvents such as chloroform, THF, and 1,4-dioxane. To our knowledge, there is relatively little research on the self-assembly of nucleobase-containing synthetic polymers in aqueous solutions.^6,13–18^ One significant report by the van Hest group synthesized poly(ethylene glycol)(PEG)-*b*-poly(nucleobase) block copolymers *via* ATRP and investigated the self-assembly behavior of a series of amphiphilic block copolymers (containing single nucleobase functionality (A or T) and the mixed A/T system) in aqueous solutions. Temperature dependent UV-vis analysis was used to confirm that A–T base pair interactions occurred upon mixing of complementary nucleobase polymers. This work indicated that in the single nucleobase system (A or T) self-assembly was governed by the hydrophobic–hydrophilic balance, however in the A/T mixed systems the complementary nucleobase interactions played a crucial role in the block copolymer assembly through shifting the hydrophobic-to-hydrophilic balance of the blocks towards increased hydrophilicity.^6^ Based on this pioneering work, our goal is to further study the aqueous self-assembly behavior of nucleobase-containing polymers (through mixing and copolymerization approaches) and exploit the effects of nucleobase interactions, self-assembly preparation methods and annealing on the resultant morphologies.

In solution, amphiphilic block copolymers can assemble into a variety of morphologies, of which the most common morphologies are spherical micelles, cylindrical micelles and vesicles.^19,20^ More complex structures have also been reported such as disk-like,^21,22^ toroidal,^23–25^ helical^26,27^ and bicontinuous micelles.^28–32^ For example, Holder and Sommerdijk *et al.* reported the formation and detailed characterization of bicontinuous micelles.^32,33^ In these reports block copolymers containing peptide,^31^ semicrystalline^29,32^ and amorphous^30^ segments were all utilized to prepare bicontinuous micelles, which were analyzed and visualized in detail by cryo-electron tomography (cryoET). In addition, the factors affecting the formation of bicontinuous micelles were also investigated, including temperature,^29^ selection of common solvent,^30^ the sequence of peptide,^31^ molecular weight distribution,^31^ and polymer composition.^30,32^ More recently, they reported that both the outer diameters and internal pore sizes of bicontinuous nanoparticles could be tuned simply by changing the initial polymer conditions and tuning the hydrophobic–hydrophilic fractions, respectively, which may allow for the development of bicontinuous nanospheres with a view towards a range of applications such as controlled release^34^ or templates for inorganic or hybrid materials.^32^

Polymers prepared from oligo(ethylene glycol) monomers are of interest in a wide range of biologically relevant applications.^35^ These polymers possess graft structures comprised of a carbon–carbon backbone and multiple oligo(ethylene glycol) side chains. Although they are not standard linear poly(ethylene glycol)s (PEG), as the oligo(ethylene glycol) chains take up a large weight fraction in the polymer structure, such polymers are still water-soluble and biocompatible in most cases.^35^ In addition, these polymers may exhibit stimuli-responsive properties, such as temperature-responsive behavior, which are not attainable with a linear PEG.^35–37^ Moreover, these polymers are easy to polymerize to prepare either homopolymers or copolymers using well-established controlled radical polymerization techniques.^35,38,39^

Here, we prepared a series of poly(oligo(ethylene glycol) methyl ether methacrylate) (POEGMA) block copolymers with the hydrophobic block of adenine, thymine or a 1 : 1 mixture of these two monomers. Self-assemblies of these nucleobase-containing block copolymers were subsequently prepared and their size and morphology were investigated in aqueous solutions. We specifically explored the effect of different good solvents in the assembly process, using DMSO and DMF which are known to suppress nucleobase H-bonding interactions yet have different abilities to solubilize the nucleobase block.^6^ This is of interest as it has been shown by Holder and Sommerdijk that the hydrophilic–hydrophobic balance as well as solvent selectivity is important in the formation of bicontinuous polymer nanospheres.^27^ This allows us to directly explore the effect of solvent on the aqueous assembly procedure for nucleobase containing polymers. This approach allowed us to tailor the resultant morphologies and, through an annealing process, allows for a change in morphology towards more complex nanostructures.

## Experimental section

### Materials

Oligo(ethylene glycol) methyl ether methacrylate (OEGMA, average *M*_n_ = 300 g mol^–1^) was bought from Aldrich and passed through a column of neutral alumina to remove the inhibitor. 2,2′-Azo-bis(isobutyronitrile) (AIBN) was purchased from Molekula and recrystallized from methanol. 2-Cyano-2-propyldodecyl trithiocarbonate (CPDT) was synthesized according to a previous report.^40^ The preparation of 3-bromopropyl methacrylate, 3-(adenin-9-yl)propyl methacrylate (AMA, Fig. S1†), and 3-(thymin-1-yl)propyl methacrylate (TMA, Fig. S2†) is according to the previous literature.^41^ *N*,*N*-Dimethylformamide (DMF), dimethyl sulfoxide (DMSO), and other solvents were used as received from Fisher. Deuterated solvents were all purchased from Apollo Scientific.

### Synthesis of POEGMA_70_ macro-CTA

OEGMA (1.8 g, 6 mmol), CPDT (17 mg, 0.05 mmol), and AIBN (1 mg, 0.006 mmol) were dissolved in 1,4-dioxane (4.5 mL). The mixture was thoroughly degassed *via* 4 freeze–pump–thaw cycles, back filled with nitrogen and then immersed into an oil bath at 65 °C for 6 hours. The reaction was quenched by immersion in a liquid nitrogen bath and exposure to air. The mixture was precipitated in diethyl ether. The resultant yellow polymer was characterized by ^1^H NMR spectroscopy in CDCl_3_ and DMF SEC (with PMMA standards). *M*_n_ (NMR) = 21.0 kDa, *M*_n_ (SEC) = 19.5 kDa; *Đ*_M_ = 1.18 (see Fig. S3†).

### Synthesis of block copolymers using POEGMA_70_ as a macro-CTA

The typical procedure is as follows: POEGMA_70_ (1 eq.), AMA (*x*), TMA (*x*), and AIBN (0.1 eq.) were dissolved in DMF or DMSO. The mixture was thoroughly degassed *via* 4 freeze–pump–thaw cycles, back filled with nitrogen then immersed into an oil bath at 60 °C. The reaction was quenched by immersion in a liquid nitrogen bath and exposure to air. The mixture was precipitated in a mixture of methanol and diethyl ether (v/v, 1 : 20) and then washed several times. The light yellow polymers (**1–3**) were dried in a vacuum oven overnight and characterized by ^1^H NMR spectroscopy in DMSO-*d*_6_ and DMF SEC (with PMMA standards) (see Table 1 and Fig. S4† for characterization data).

**Table 1:** Characterization data for the macro-CTA and block copolymers **1–3**

Polymer	*M*_n,th_ (kDa)	*M*_n,NMR_ ^*a*^ (kDa)	*M*_n,SEC_ ^*b*^ (kDa)	*Đ*_M_	*m* ^*d*^	*f* ^*d*^
POEGMA_70_	20.5	21.0	19.5	1.18		
POEGMA_70_-*b*-PAMA_*m*_, **1**	47.1	48.9	—^*c*^	—^*c*^	104	0.43
POEGMA_70_-*b*-PTMA_*m*_, **2**	46.2	47.2	34.8	1.41	103	0.44
POEGMA_70_-*b*-(PAMA_0.5_-*co*-PTMA_0.5_)_*m*_, **3**	46.7	47.3	31.4	1.37	102	0.44

^*a*^Determined by ^1^H NMR spectroscopy.

^*b*^Determined by SEC analysis (DMF as the eluent, PMMA standards).

^*c*^Polymer is not fully soluble in DMF.

^*d*^Calculated from *M*_n, NMR_, *m*: DP of the nucleobase block, *f*: POEGMA weight fraction in the copolymer.

### Self-assembly

Polymers **1–3** were self-assembled using a solvent switch method. The polymer was dissolved in DMF or DMSO (at 8 mg mL^–1^) and stirred for 2 days. After this time an excess of 18.2 MΩ cm water was added by using a syringe pump at a rate of 1 mL h^–1^. The final volume ratio between water and organic solvent was 8 : 1. The solution was then dialyzed against 18.2 MΩ cm water, incorporating at least 6 water changes, to afford self-assemblies of *ca.* 1 mg mL^–1^.

### NMR spectroscopy

^1^H NMR and ^13^C NMR spectra were recorded on a Bruker DPX-300 or DPX-400 spectrometer with DMSO-*d*_6_ or deuterated chloroform (CDCl_3_) as the solvent. The chemical shifts of protons were reported relative to tetramethylsilane at *δ* = 0 ppm when using CDCl_3_ or solvent residues (DMSO ^1^H: 2.50 ppm).

### Size exclusion chromatography

Size exclusion chromatography (SEC) was obtained in HPLC grade DMF containing 5 mM NH_4_BF_4_ at 50 °C, with a flow rate of 1.0 mL per minute, on a set of two PLgel 5 μm Mixed-D columns, plus one guard column. SEC data were analyzed with Cirrus SEC software calibrated using poly(methyl methacrylate) (PMMA) standards. The SEC was equipped with both differential refractive index (DRI) and UV detectors.

### Transmission electron microscopy

Transmission electron microscopy (TEM) observations were performed on a JEOL 2000FX electron microscope at an acceleration voltage of 200 kV. All TEM samples were prepared on graphene oxide (GO)-coated carbon grids (lacey carbon or Quantifoil R2/2) which allows high contrast TEM images to be acquired without staining.^42^ Generally, a drop of sample (20 μL) was pipetted on a grid, blotted immediately and left to air dry.

Cryogenic transmission electron microscopy (cryo-TEM) was performed on a Tecnai G2 12 Twin TEM equipped with a Gatan CCD camera at an acceleration voltage of 120 kV. The temperature of the cryo stage was maintained below –170 °C during imaging. For sample preparation, 5 μL of the sample was deposited onto a lacey carbon grid, blotted immediately and vitrified by plunging into liquid ethane.

### Small-angle X-ray scattering

Measurements were performed at the Australian Synchrotron facility on the SAXS/WAXS beamline at a photon energy of 12 keV. The samples in aqueous solution were collected at sample-to-detector distances of 7160, 3252, and 727 mm to give a *q* range from 0.0023 to 1.2 Å^–1^. The scattering from a blank was measured in the same location as the sample collection and was subtracted for each measurement. Data were normalized for total transmitted flux using a quantitative beamstop detector and absolute scaled using water as an absolute standard. The two-dimensional isotropic SAXS images were converted into one-dimensional SAXS scattered intensity profiles (*I*(*q*) *vs*. *q*) by circular averaging using the software package ScatterBrain developed at the Australian Synchrotron. The profiles from the different sample-to-detector distances were merged using Primus^43^ and were analyzed using the NCNR package in IGOR Pro.^44^

### Light scattering

Hydrodynamic diameters (*D*_h_) and size distributions of the self-assemblies were determined by dynamic light scattering (DLS). The DLS instrumentation consisted of a Malvern Zetasizer NanoS instrument operating at 25 °C with a 4 mW He–Ne 633 nm laser module. Measurements were made at a detection angle of 173° (back scattering), and Malvern Zetasizer 7.03 software was used to analyze the data.

Static light scattering (SLS) measurements were conducted with an ALV CGS3 (*λ* = 632 nm) at 20 °C. The data were collected from 12° up to 30° with an interval of 2° and from 30° up to 150° with an interval of 10°, calibrated with filtered toluene and filtered water as backgrounds. The refractive index (RI) of the polymer self-assembly in water was measured to be 0.13 mL g^–1^.

### UV-vis spectroscopy

UV-vis spectroscopy was carried out on a Perkin Elmer Lambda 35 UV/vis spectrometer, equipped with a PTP-1 + 1 Peltier temperature programmer and stirring system, and a PCB 1500 water system to maintain the desired temperature throughout the experiments. Quartz cuvettes were used for all the experiments.

### Differential scanning calorimetry

Differential scanning calorimetry (DSC) measurements were performed on a Mettler Toledo HP DSC827 from – 90 to 180 °C with a heating rate of 10 °C min^–1^ and a cooling rate of 20 °C min^–1^. Data were analyzed using Mettler Toledo STARe software v9.20. Glass transition temperatures (*T*_g_) were taken as the peak of the inflection tangent.

## Results and discussion

### Synthesis of macro-CTA and nucleobase diblock copolymers

**Scheme 1:**
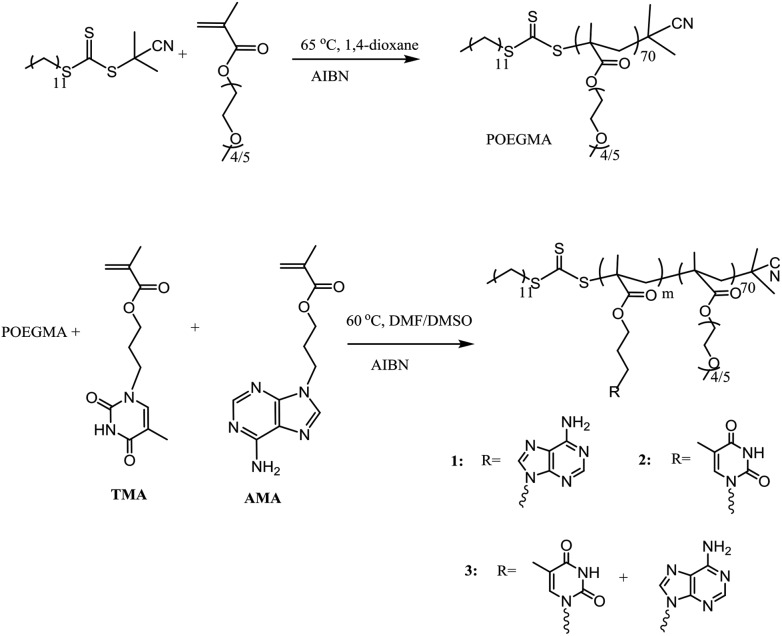
Synthetic route for the preparation of POEGMA_70_ and the nucleobase-containing block copolymers, **1–3**.

A series of nucleobase diblock copolymers were prepared as shown in Scheme 1. Namely, POEGMA was synthesized by RAFT polymerization, using 2-cyano-2-propyldodecyl trithiocarbonate (CPDT) as the CTA, AIBN as the initiator (CTA : AIBN = 1 : 0.1), and 1,4-dioxane as the solvent. The monomer conversion for the polymerization was 57%, as determined by ^1^H NMR spectroscopy. The degree of polymerization (DP) of the purified POEGMA was *ca.* 70, determined by ^1^H NMR spectroscopy by comparing the integration of the signal from CPDT (*δ* = 3.2 ppm) with those from the backbone of the polymer (*δ* = 4.10 ppm). Furthermore, SEC was used to characterize the molecular weight and molecular weight distribution of POEGMA_70_ and revealed a narrow molecular weight distribution (*Đ*_M_ = 1.18) (Fig. S3† and Table 1). In addition, the DRI and UV (309 nm, from the trithiocarbonate end group) traces overlap well, indicating good end group fidelity. POEGMA should exhibit temperature-responsive behavior and generally displays a lower critical solution temperature (LCST), which has been widely reported in previous reports.^35^ Indeed, the cloud point of the macro-CTA POEGMA_70_ was found to be *ca.* 65 °C, which was consistent with the values reported in the literature.^35^

To synthesize nucleobase-containing block copolymers, RAFT polymerizations, using POEGMA_70_ as the macro-CTA, were performed in DMF or DMSO, using AMA (polymer **1**), TMA (polymer **2**) or a 1 : 1 mixture of AMA and TMA (polymer **3**), as monomers. The molar ratio of POEGMA_70_ : monomer : AIBN was kept at 1 : 100 : 0.1 in all polymerizations. High conversion (≥99%) was attained for each polymerization. The characterization data for all the block copolymers are shown in Table 1. It should be noted that the DP of the resultant nucleobase block was *ca.* 100 for each polymer, which was determined by ^1^H NMR spectroscopy by comparing the integrals of signals from the nucleobase block (*δ* = 8.16–8.10 ppm (for AMA), 7.50–7.30 ppm (for TMA)) with those from the POEGMA block (*δ* = 3.28–3.23 ppm). In addition, the POEGMA weight fraction in the copolymer (*f*) was calculated using the molecular weight determined by ^1^H NMR spectroscopy (*M*_n, NMR_). Moreover, SEC traces of the macro-CTA and the synthesized block copolymers were overlaid (Fig. S4†), where a clear shift in molecular weight distribution suggested successful chain extension. However, it should be noted that the resultant block copolymers have relatively poor solubility in DMF, especially polymer **1**, POEGMA_70_-*b*-PAMA_104_, and thus the SEC analysis of **1** using DMF as the eluent was not possible. Furthermore, the thermal properties of polymers **1–3** were investigated (Fig. S4†). Peaks at *ca.* – 41 °C and 130–150 °C were correlated to the glass transition temperature (*T*_g_) of POEGMA and the nucleobase-containing block, respectively.^45^ The peak at *ca.* 60 °C might be a consequence of incomplete microphase separation with both POEGMA and nucleobase-containing blocks coexisting in the copolymer.^46^

### Self-assembly of polymers **1–3**

**Fig. 1:**
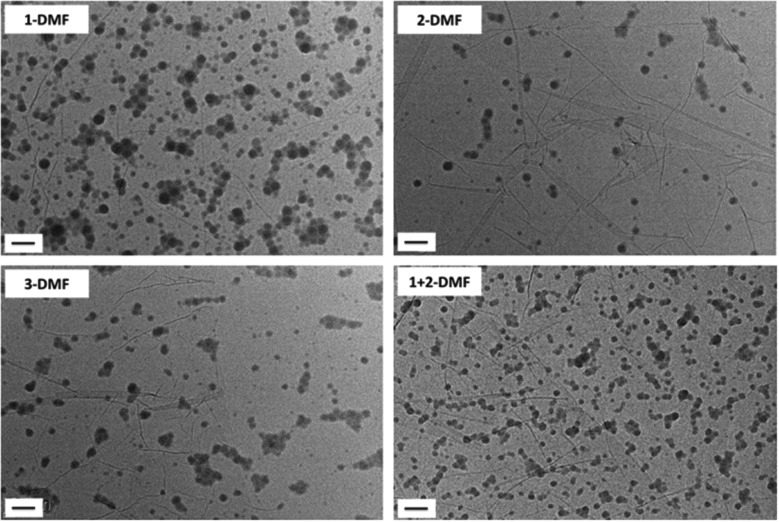
Representative dry-state unstained TEM images on GO grids of self-assemblies **1** **-DMF**, **2** **-DMF**, **3** **-DMF** and **1** + **2** **-DMF**. Scale bar: 100 nm.

The solvent-switch method was applied to prepare nucleobase-containing self-assemblies in water as it is considered a suitable approach to prepare *crew-cut* self-assemblies.^47^ DMF was first selected as the common solvent as both the POEGMA and nucleobase-containing blocks were relatively soluble in this solvent, and water was utilized as the selective solvent for the POEGMA block. Furthermore, it has been shown that A–T H bonding interactions are very weak in DMF and hence the resultant assemblies are expected to be determined based on their hydrophilic : hydrophobic balance.^48^ Indeed, for this series the same morphology was predicted as all have a hydrophilic weight fraction of *ca.* 40%. A series of self-assemblies were prepared from polymers **1**, **2**, **3** and a 1 : 1 mixture of **2** and **3** (**X-DMF**, where **X** is the polymer name: **1**, **2**, **3**, and **1** + **2**). The initial polymer concentrations in the common solvent were fixed at 8 mg mL^–1^. Water was added to the solution at a rate of 1 mL h^–1^ until the final volume ratio between water and DMF was 8 : 1. The solutions were then dialyzed against 1 L water and the final assemblies formed had concentrations *ca.* 1 mg mL^–1^. The 4 solutions were then diluted to 0.2 mg mL^–1^ before being characterized by TEM and DLS analysis. DLS analysis of the solutions indicated that the assemblies were well-defined (with a size *ca.* 50 nm) with narrow size distributions (PD < 0.18) (Fig. S5†). By dry-state TEM analysis only spherical micelles were observed of *ca.* 30 nm (Fig. 1). It should be noted that the unstained TEM images were prepared on graphene-oxide (GO)-coated TEM grids and folds were usually observed in the backgrounds.^42^ Synchrotron small-angle X-ray scattering (SAXS) studies were performed on the final assemblies to confirm the DLS and TEM data. In all four cases, a main population of core–shell spherical micelles was observed in solution with the total diameter of the micelles consistent with the TEM data (Fig. S6 and Table S1,† a small population of spherical micelles is also fitted to enable a better fit at high *q* values). The SAXS diameters are slightly smaller than the DLS values, this is due to the SAXS model used assuming the shell has a uniform density of polymeric chains (Table S1†). Actually, more solvation of the chains occurs at the water–micelle interface compared to the core–shell interface. This implies that the density of the shell is not uniform and the outer shell is likely to be too hydrated to provide enough contrast for SAXS analysis. The formation of spherical micelles is not expected based on the hydrophilic weight fraction of the block copolymer. However, given that the solubility parameter of DMF (*δ* = 24.8)^49^ is proposed to be much larger than the one of the nucleobase polymers, this would lead to a low amount of DMF being present in the core and hence a lower aggregation number (*N*_agg_) at the onset of micellization.^17^ Therefore, the low *N*_agg_ facilitates the formation of spherical micelles as opposed to the expected vesicular morphology.^50^

According to previous studies, the selection of the common solvent influences the morphology of the aggregates as different common solvents can change the relative coil dimensions of the core and coronal chains.^20,30,47^ DMSO was observed to be a better solvent for nucleobase-containing polymers than DMF as it is an extremely good hydrogen-bonding acceptor and has a high electron donating capacity.^51,52^ Therefore, DMSO was expected to increase the solubility and stretching of the polymer chains, which may in turn affect the resultant morphologies. Self-assemblies in DMSO were prepared by a similar procedure to that described using DMF as a good solvent, with the final concentration of all 4 solutions *ca.* 1 mg mL^–1^. Self-assemblies of **1**, **2**, **3** and a 1 : 1 mixture of **1** and **2** were diluted to 0.05 mg mL^–1^ before being characterized by TEM, DLS, and SAXS analysis (Fig. S7 and S8, and Table S2,† **X-DMSO**, where **X** is the polymer number).

**Fig. 2:**
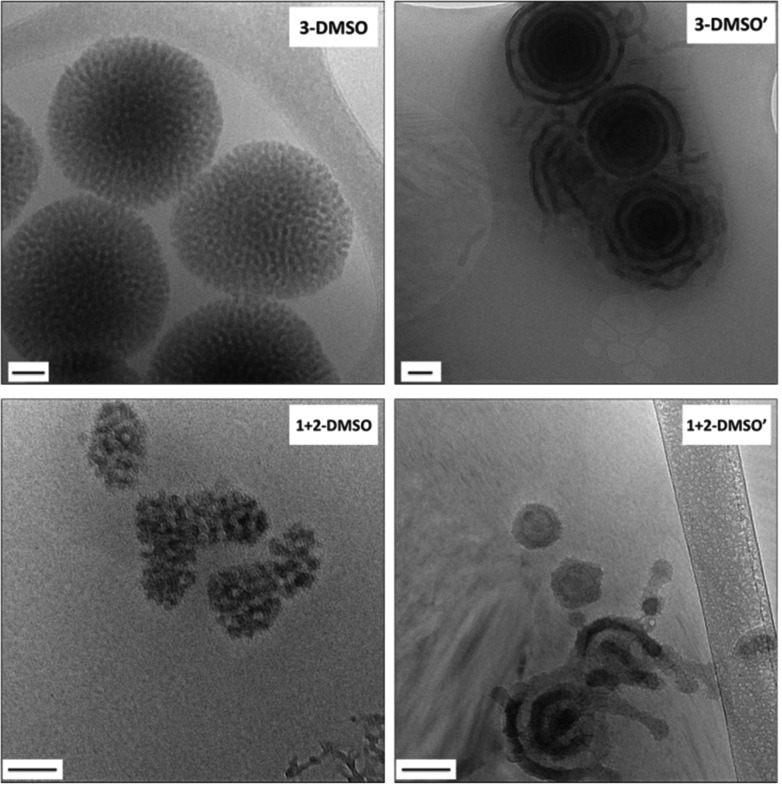
Representative cryo-TEM images of self-assemblies **3** **-DMSO** with a concentration of 1 mg mL^–1^ and its annealed sample **3** **-DMSO′** at 0.2 mg mL^–1^; **1** + **2** **-DMSO** at a concentration of 1 mg mL^–1^ and its annealed sample **1** + **2** **-DMSO′** at 0.2 mg mL^–1^. Scale bar: 100 nm.

**Fig. 3:**
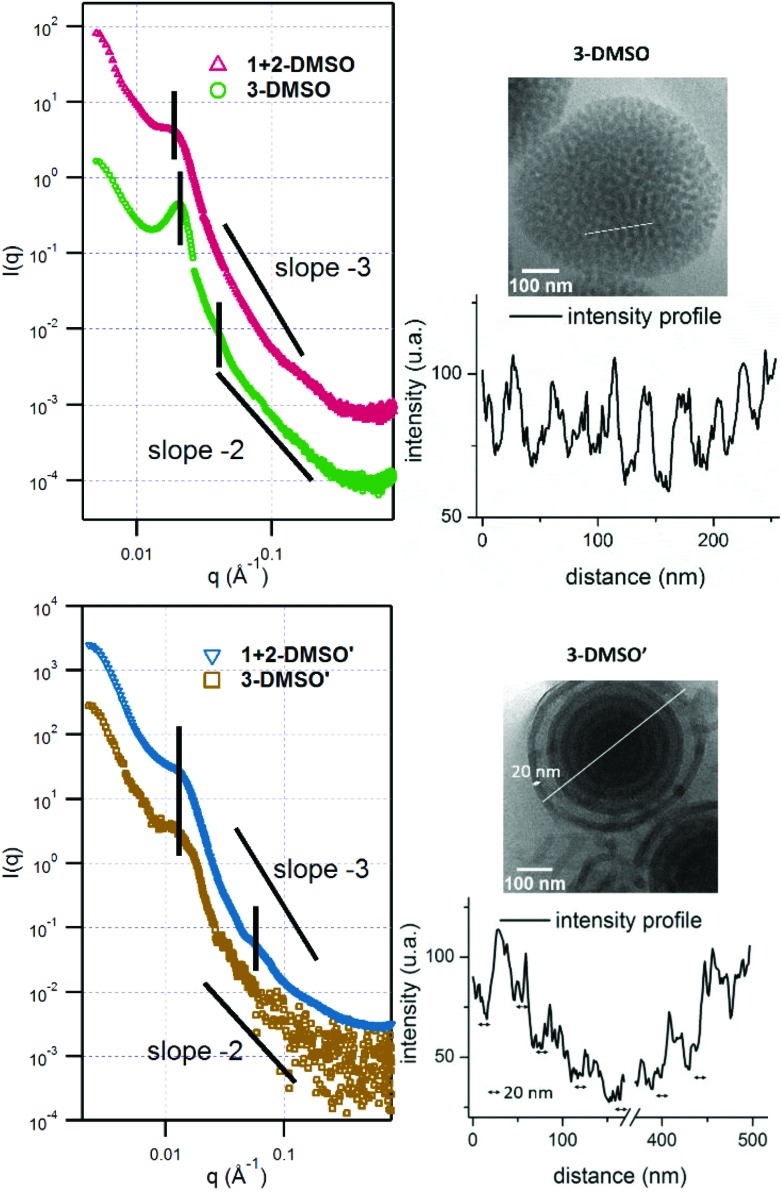
SAXS profiles of self-assemblies **3** **-DMSO**, **1** + **2** **-DMSO**, **3** **-DMSO′**, and **1** + **2** **-DMSO′**. The vertical black lines indicate the major peaks, indicative of the internal structure of the assemblies. The **1** + **2** **-DMSO** and **1** + **2** **-DMSO′** curves have been shifted by a factor 10 to improve clarity. Cryo-TEM images of **3** **-DMSO** and **3** **-DMSO′** and the corresponding intensity profile along the white line on the TEM images.

When the self-assembly of polymer **1**, POEGMA_70_-*b*-PAMA_104_ was explored, as described above, precipitation occurred upon addition of water, indicating that no stable assemblies could be formed in this system. Further attempts to optimize this assembly were unsuccessful and this could be attributed to the formation of adenine : adenine interactions during the assembly process which leads to precipitation of the polymer. However, the self-assembly of polymer **2**, POEGMA_70_-*b*-PTMA_103_ led to the formation of small clustered structures (Fig. S7,† **2** **-DMSO**), which unexpectedly possessed one population by DLS analysis (*ca.* 200 nm, PD = 0.24). SAXS analysis confirmed the presence of core–shell spherical micelles (Fig. S8 and Table S2†). Interestingly, for polymer **3** by dry-state TEM analysis spherical bicontinuous micelles were observed upon assembly in DMSO (Fig. S7,† **3** **-DMSO**). The sample was further imaged by cryo-TEM at different concentrations, 1 mg mL^–1^ and 0.2 mg mL^–1^ (Fig. 2, **3** **-DMSO** and Fig. S9,† respectively), with the self-assemblies appearing to have the same morphology and size (*ca.* 400 nm) at these two concentrations, indicating that dilution had no effect on the morphology. By DLS analysis, the size distribution of the particles was slightly broadened (PD = 0.29) with two populations observable. The spherical shape of the assemblies is confirmed by the bell-like shape of the Kratky plot^53,54^ of the SAXS profile (Fig. S10†). The log–log SAXS profile exhibits two major broad peaks at *q* values of 0.021 and 0.041 Å^–1^ that correspond to periodic values of 30.3 and 15.3 nm, respectively. These two values confirm the bicontinuous network obtained by TEM given that a bicontinuous phase can usually be described in terms of interwoven networks.^33^ One network is generally made of a bilayer structure while the other one is a film of “opposite” polarity material. The two characteristic distances observed are likely to be the width of one network and the width of two consecutive networks. This was calculated by the careful analysis of the intensity profile of the cryo-TEM image giving distances of 30.5 nm between two consecutive distances of maximum intensity (white pixels on TEM image), and 13.1 nm for the distance around the maximum intensity at about half the maximum intensity, Fig. 3). To explore the effect of the complementary nucleobase interactions on the assembly behavior further, we explored the self-assembly of a 1 : 1 mixture of polymers **1** and **2**. In this case bicontinuous micelles were also observed (Fig. 2, **1** + **2** **-DMSO**), although they were smaller in size (*ca.* 150 nm) and not as spherical compared to those obtained from copolymer **3**. These particles had a narrow size distribution (PD = 0.083) with only one population of *ca.* 181 nm by DLS analysis (Fig. S7,† **1** + **2** **-DMSO**). A Guinier plot^55,56^ of the SAXS profile of **1** + **2** **-DMSO** gives an *R*_g_ value of 39.5 nm. This value is much smaller than the radius determined by TEM and DLS, likely indicating that there is a large amount of water trapped in the assemblies. A spherical shape for the assemblies is confirmed by a Kratky plot of the SAXS profile (Fig. S10†). The SAXS profile is characterized by one broad peak with a maximum intensity at a *q* value of 0.019 Å^–1^ which corresponds to a characteristic distance of 32.8 nm (Fig. 3). A weaker peak is also observed, almost in the background, at a *q* value of 0.228 Å^–1^, or a periodic distance of 2.8 nm. A similar analysis of the TEM images gives a periodic distance of 31.8 nm between two lower contrast areas, which confirms the repeat unit value from the SAXS analysis. The Porod region (*q* > 0.2 Å^–1^) yields information about the “fractal dimension” of the scattering objects.^57^ A linear slope of almost –3 is observed for **1** + **2** **-DMSO**, while a value of –2.2 is obtained for **3** **-DMSO**. Values between –2 and –3 are typically observed for mass fractals such as branched systems or networks, which confirms the morphology of the two samples. A value closer to –2 indicates a smoother surface, or smaller rough patches: such values are in accordance with the TEM images (Fig. 2).

Interestingly, different morphologies were observed when assemblies were performed in different common solvents: (DMF and DMSO). It should be noted that the weight fraction (*f*) of the POEGMA block in polymers **1–3** was *ca.* 0.40 (Table 1). According to the rule of hydrophilic weight fraction (*f*_hydrophilic_) for predicting resultant morphologies reported by Discher and Eisenberg,^58,59^ vesicle or cylinder structures were expected to form from polymers **1–3**. However, in this study, only spherical micelles (*f*_hydrophilic_ > 50%) were observed when using DMF as the common solvent as a result of the difference in solubility parameters between the good solvent and hydrophobic block. However, the solubility parameter of DMSO (*δ* = 12.0) is proposed to be similar to that of the nucleobase polymer and hence would expect to lead to higher *N*_agg_s during assembly and thus may account for the formation of more complex morphologies. Such effects on self-assembly are known and examples in the literature include sensitivity to chain chemistry, molecular weight and chain structure.^59^

We have observed that nucleobase-containing polymers are more soluble in DMSO than DMF, which would lead to a higher degree of stretching of the core-forming blocks, thus affecting the balance between hydrophobic domains and hydrophilic corona chains.^20,30,47,60^ Therefore, polymer curvatures in DMF were expected to be smaller than those in DMSO due to different polymer–solvent interactions, which in turn resulted in different morphologies. Such a difference in the curvature is observed for polymer **2** with a diameter of 35 nm in DMF and 40 nm in DMSO (determined by SAXS, Table S2†). Moreover, a smaller scattering length density is obtained for **2** **-DMSO** compared to the value for **2** **-DMF** which correlates to a lower density of the chains in the micellar core in DMSO than in DMF and thus a higher degree of stretching of the core-forming block TMA. In addition, the viscosity of DMSO (*η* = 2.0 Ns m^–2^, 25 °C) is significantly higher than in DMF (*η* = 0.80 Ns m^–2^, 25 °C) and therefore leads to slower water–organic phase mixing and a lower precipitation rate, which would be favorable to the formation of larger nanoparticles.^61^ To this end, the formation of DMSO–polymer droplets in an aqueous environment is conceivable and this we suggest would favor the formation of bicontinuous structures. This is consistent with previous proposals on the formation of bicontinuous micelles (*i.e.* bicontinuous micelles originate from polymer-rich good solvent droplets and the exchange of good-solvent with water leading to microphase separation and eventually the final morphology).^31,32^

It should also be noted for the DMSO assemblies that the composition of nucleobases in the polymers affects the morphology of the resultant self-assemblies. Although polymers **1–3** possessed a similar weight fraction of POEGMA, different aggregation behaviors were observed when different nucleobase functionalities were present in the polymers (Fig. S7†). When adenine and thymine were both present in the system (either as a pure copolymer or a mixture of 2 complementary homopolymers), micelles with internal structures were observed (Fig. 2, **3** **-DMSO** and **1** + **2** **-DMSO**). In contrast, for the thymine-containing polymer **2**, which possessed relatively weak thymine–thymine interactions, particles with no internal structure were observed. Thus, it appears that the difference in nucleobase interactions rather than the weight fraction plays a key role in determining the resultant morphology. This observation is consistent with the related literature where the amino acid sequence in peptides, rather than weight fraction, resulted in different aggregation behaviors.^31^ In addition, it was observed that the particles **3** **-DMSO** and **1** + **2** **-DMSO** (and their annealed analogues) were still stable after *ca.* 6 months (Fig. S11†), while **2** **-DMSO** appeared to reorganize into smaller nanostructures (Fig. S12†). This observation indicated that particles containing both adenine and thymine have improved long term stability compared to those possessing only one nucleobase functionality.

### Effect of annealing on the self-assembly

Annealing is a common method for the formation of well-defined microphase separated block copolymers^62–64^ and the self-assembly of DNA.^65–67^ Annealing involves a heat treatment and a cooling process, where heating mobilizes the polymer chains and cooling can refine the resultant interactions and structures. Herein, we took advantage of the annealing method and applied it to our solution self-assemblies. The annealing experiments were performed on a variable-temperature UV-vis spectrometer (*λ* = 500 nm). It should be noted that the samples had no absorption in the wavelength of visible light, determined by both UV spectroscopy and visual inspection and therefore this did not affect the samples.

Annealing was firstly applied to 1 mg mL^–1^ solutions of **1** **-DMF**, **2** **-DMF**, **3** **-DMF**, and **1** + **2** **-DMF** to investigate the effects of annealing (resultant solutions named as **1** **-DMF′**, **2** **-DMF′**, **3** **-DMF′** and **1** + **2** **-DMF′** respectively). After annealing (annealing conditions: 15–85 °C and then 85–15 °C with a rate of 1 °C min^–1^ for 3 cycles) the spherical structures were maintained and no morphology transition was observed. However, the micelles possessed narrower size distributions (PD < 0.1) compared to the assemblies before annealing, as observed by both TEM, DLS and SAXS analysis (Fig. S13 and S14†). These results suggested that the annealing process enabled a reorganization of the polymer chains to form a better-defined structure.

The effect of annealing on the resultant morphologies prepared by the solvent-switch method using DMSO as the common solvent was also investigated. Annealing was applied to 0.2 mg mL^–1^ solutions of **2** **-DMSO**, **3** **-DMSO**, and **1** + **2** **-DMSO** (named as **X-DMSO′**, where **X** is the polymer number). The samples were diluted before being characterized by TEM, DLS, and SAXS analysis (Fig. 2 and Fig. S15 and S16†). By DLS analysis it was found that all the annealed samples possessed narrower size distributions than those before annealing (PD 0.24, 0.29, 0.083 *vs*. 0.14, 0.17, 0.068). In addition, by TEM analysis morphology changes were observed for all the samples. Vesicles were observed upon annealing the small clustered assemblies (**2** **-DMSO)** prepared from polymer **2** (Fig. S15,† **2** **-DMSO′**, *D*_h_ = 163 nm). The *R*_g_ obtained from the SAXS profile is around 75 nm (from the Kratky–Porod plot, Fig. S16†). The *ρ* parameter (*R*_g_/*R*_h_) thus is close to 1, which indicates a vesicle morphology in solution, and confirms the morphology observed by TEM. Two broad peaks can be seen on the SAXS profile at *q* values of maximum intensity of 0.0111 and 0.034 Å^–1^ or characteristic distances of 56.6 and 18.3 nm (Fig. S16†). Moreover, the Kratky–Porod plot^68^ allows the determination of the thickness of the shell, which is found to be 27 nm (Fig. S16†). For the mixed A-*co*-T copolymer **3**, after annealing, vesicular structures were observed by dry-state TEM, some of which appeared with multiple layers (multilamellar vesicles, Fig. S15,† **3** **-DMSO′**). These onion-like structures were further confirmed by cryo-TEM (Fig. 2, **3** **-DMSO′**), which were visibly different from the self-assemblies before annealing (Fig. 2, **3** **-DMSO**). The SAXS profile exhibited one broad peak at *q* = 0.0237 Å^–1^ or *d* = 49.5 nm, which is likely to account for two consecutive layers (one dark and one white on the cryo-TEM image, Fig. 3). The dispersity of the white layer is quite high according to the TEM images while the thickness of the dark layer is well controlled and is about 20 nm, as determined by the intensity profile of the cryo-TEM images (Fig. 3). For a 1 : 1 mixture of **1** and **2**, hollow structures with single or multiple layers were observed (Fig. 2 and Fig. S15,† **1** + **2** **-DMSO′**, *D*_h_ = 168 nm), suggesting that there was a solid-to-hollow transition induced by annealing in these mixed A/T systems. The SAXS profile of **1** + **2** **-DMSO′** is similar to that of **3** **-DMSO′** (Fig. 3) and exhibits the same broad peak at low *q* values. Another peak is visible at higher *q* values, as the profile is not as noisy, at *q* = 0.0590 Å^–1^ or *d* = 10.6 nm. For both **3** **-DMSO′** and **1** + **2** **-DMSO′**, an *R*_g_ of 70–75 nm was calculated, however this value has to be taken with some caution as the limit of the *q* range is reached. To prove that the polymers were stable to the annealing conditions, solutions before (**3** **-DMSO)** and after annealing (**3** **-DMSO′)** were freeze-dried and the obtained polymers (**3** and **3′**) were then characterized by both SEC analysis and ^1^H NMR spectroscopy. No obvious differences in molecular weight and molecular weight distribution were observed for **3** before and after annealing (Fig. S17†). In addition, the ^1^H NMR spectra of the polymers harvested before and after annealing were identical to spectra before self-assembly (Fig. S17†). These results confirm that the morphology reorganization was not a result of changes in the polymer structure but instead was an effect of reorganization induced by the nucleobase functionality interactions during the annealing process. Indeed, the difference in assembly behavior upon annealing in DMSO compared to DMF may be explained by the greater solubility of the nucleobase block in DMSO (as well as stronger nucleobase interactions) which in turn enables a more pronounced reorganization upon annealing to form a better defined construct.

Sommerdijk reported that the same polymer (poly(ethylene oxide)-*b*-poly(*n*-butylmethacrylate)) formed either bicontinuous micelles or multilamellar vesicles depending on the nature of the good solvent in the self-assembly process.^28^ In this study these two morphologies, bicontinuous micelles and multilamellar vesicles, were formed from one polymer (*e.g.*, polymer **3**, POEGMA_70_-*b*-(PAMA_0.5_-*co*-PTMA_0.5_)_102_), however different morphologies were observed before and after annealing. According to the previous literature, the formation of these morphologies resulted from the different polymer curvatures resulting from changes in the solubility parameter for the core forming block.^30^ These observations indicated that the apparent polymer dimensions before and after annealing might be altered, leading to different resultant morphologies. We thus assume that annealing results in chain rearrangement (through breaking and reforming complementary nucleobase interactions), which decreases the polymer curvature and results in a bicontinuous-to-lamellar transition as a consequence of an increase in hydrophilic fraction.^29^ Such an observation correlates well with previous observations by van Hest, in which the presence of complementary nucleobase functionality shifted the amphiphilic balance towards increased hydrophilicity.^6^

### Effect of annealing conditions

Annealing conditions and their effects on the resultant morphologies were investigated. The annealing conditions used above included 3 heating–cooling cycles, where a solution was heated from 15 °C to 85 °C and then cooled from 85 °C to 15 °C at a rate of 1 °C min^–1^. The annealing rate and cooling time were assumed to affect the resultant morphologies. Indeed, it is suggested that a slow annealing rate is key for the formation of well-defined structures and for the observed morphology transitions. To prove this hypothesis, **3**-**DMSO** (present as bicontinuous micelles), prepared from polymer **3**, POEGMA_70_-*b*-(PAMA_0.5_-*co*-PTMA_0.5_)_102_ at a concentration of 0.2 mg mL^–1^, was heated at 85 °C for 45 min and then cooled down slowly to 30 °C in an oil bath for *ca.* 60 min. The resultant solution was diluted to 0.05 mg mL^–1^ and then characterized by TEM and DLS analysis (Fig. S18†) with poorly-defined sheet-like structures observed rather than the original bicontinuous micelles or expected multilamellar vesicles. This result suggested that the slow heating and cooling rates play a key role in the formation of well-defined structures. This would be expected based on the proposed reorganization of the complementary nucleobases to form duplex-like polymer structures. To confirm this UV-vis analysis of **3** **-DMSO** following each annealing cycle was undertaken (Fig. S19†). The reduction in absorption maxima at 269 nm and a shift to 266 nm confirm the enhancement of complementary nucleobase interactions.

**Fig. 4:**
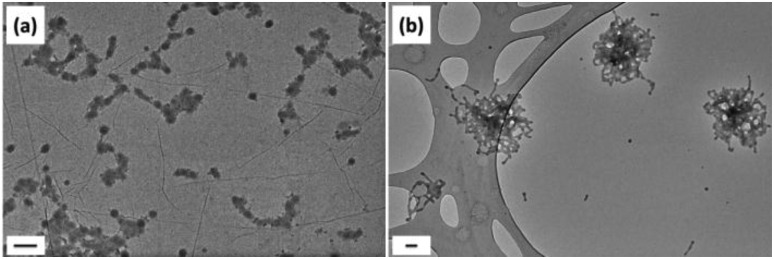
Representative unstained dry-state TEM images on GO grids of **3** **-DMSO** annealed by different methods: (a) sample was heated at 60 °C for 45 min and then cooled down in an oil bath; (b) sample was heated from 15 °C to 60 °C and then cooled down from 60 °C to 15 °C at a rate of 1 °C min^–1^ and repeated 3 times in total. Scale bar: 100 nm.

In addition, the effect of annealing temperature was also investigated. As POEGMA had a cloud point *ca.* 65 °C, the annealing temperature was set to 60 °C for comparison. Thus, bicontinuous micelles, **3** **-DMSO** with a concentration of 0.2 mg mL^–1^, were annealed using two methods: (a) the solution was heated at 60 °C for 45 min and then cooled down in an oil bath naturally (*ca*. 50 min, from 60 °C to 30 °C, non-constant rate); (b) the solution was heated from 15 to 60 °C and then cooled down from 60 to 15 °C at a rate of 1 °C min^–1^, which was repeated 3 times in total. Regardless of the annealing method the observed morphology was no longer bicontinuous micelles. Instead small aggregates were observed in the sample annealed using method (a) (Fig. 4a). When method (b) was applied, where the annealing rate was consistent with the previous annealing procedure (1 °C min^–1^), chrysanthemum-like aggregations were observed which possessed a few cylindrical tentacles (Fig. 4b). This observation further suggested that bicontinuous micelles were not the most thermodynamically favorable structure and were disassembled with annealing. Indeed, these observations indicate that the nucleobase-containing blocks can become mobile at 60 °C, however, the thermodynamically favorable onion-like structures were not formed as the annealing temperature was not high enough.

### Effect of annealing cycles on morphologies

**Fig. 5:**
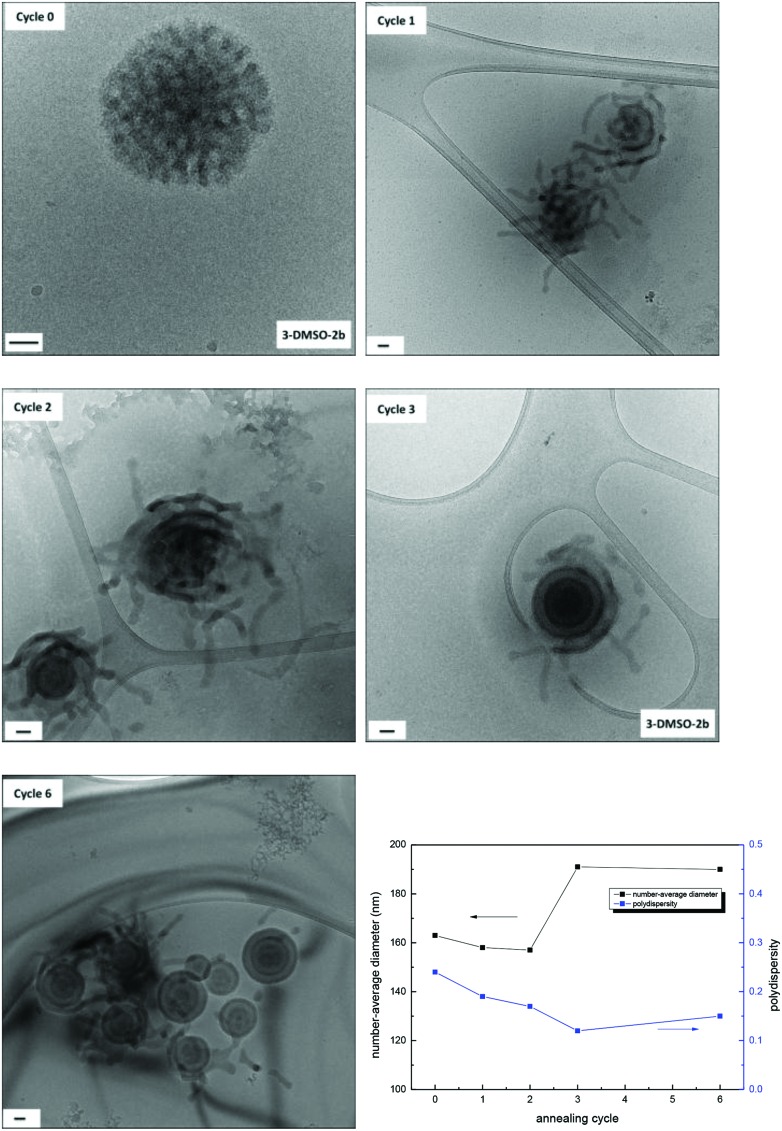
Evolution of self-assembly from **3** **-DMSO** to **3** **-DMSO′** with 6 annealing cycles characterized by cryo-TEM analysis (scale bar = 50 nm) and the number-average diameter and size distributions determined by DLS analysis.

For polymer **3**, three annealing cycles were performed to achieve the morphology transition from bicontinuous micelles to multilamellar vesicles. Herein, the effect of the number of annealing cycles on morphologies was investigated for the solution **3**-DMSO at a concentration of 0.2 mg mL^–1^. The solutions obtained after each annealing cycle were characterized by dry-state TEM (Fig. S20†), cryo-TEM and DLS analysis (Fig. 5). It was found that after the first annealing cycle the bicontinuous micelles (Fig. 5, cycle 0) were rearranged into particles with tentacles, some of which still possessed a bicontinuous structure in the center of the particles (Fig. 5, cycle 1), indicating that the mobility of the polymer chains was increased and the onset of the morphology transition. After the second annealing cycle, the particles were rearranged further and the tentacles started to fuse into layers. Finally, vesicle structures with multiple layers were formed after the third annealing cycle (Fig. 5, cycle 3). If the multilamellar vesicles were annealed for a further 3 cycles, no obvious change was observed (Fig. 5, cycle 6), indicating that the multilamellar vesicles were the final thermodynamically favorable structure in this annealing process. The SAXS profiles only exhibit partial information as the size of the morphologies cannot be determined (Fig. S21†). It is however possible to observe slight changes in the nanostructures, which confirms the changes observed by TEM.

As we proposed above, the bicontinuous micelles were induced by the nucleobase interactions within the system that were kinetically frozen upon addition of water. With annealing, the polymer chains became relatively mobile and a structural rearrangement was induced, which resulted in a change of polymer curvatures and a morphology change.

### Effect of polymer concentration

In the previous assemblies, the initial polymer concentration was 8 mg mL^–1^ in the common solvent. Herein, polymer solutions in DMSO with a lower initial concentration (2 mg mL^–1^) were prepared and the effect of concentration and water content on the resultant morphologies was investigated. Polymers **1**, **2**, **3** and a 1 : 1 mixture of **1** and **2** were dissolved in DMSO at a concentration of 2 mg mL^–1^. Water was then added to the solution at a rate of 1 mL h^–1^ until the final volume ratio between water and DMSO was 8 : 1. The resultant solutions were dialyzed to remove DMSO and the final concentrations were estimated to be *ca.* 0.2 mg mL^–1^. The solutions (**X-DMSO-2a**, where **X** is the polymer number) were then diluted to 0.05 mg mL^–1^ and characterized by TEM and DLS analysis (Fig. S22 and S23†). In general, similar structures were observed compared to **X-DMSO** which had higher initial polymer concentrations. Interestingly, for polymer **3**, POEGMA_70_-*b*-(PAMA_0.5_-*co*-PTMA_0.5_)_102_, bicontinuous micelles were observed by TEM analysis (Fig. S22,† **3** **-DMSO-2a**) although the particles possessed smaller sizes (155 nm) compared to micelles prepared from **3** **-DMSO** (421 nm). A similar observation was made for the assemblies prepared from the 1 : 1 mixture of **1** and **2**, where bicontinuous micelles of *ca.* 97 nm were observed (**1** + **2** **-DMSO-2a**), which had a similar elongated shape to **1** + **2** **-DMSO** yet were smaller in size (**1** + **2** **-DMSO**, 181 nm). These results showed that the initial polymer concentration had little effect on the resultant morphologies but affected the sizes of the particles. This can be rationalized based on the proposed mechanism of bicontinuous micelle formation:^33^ the production of polymer-rich droplets in good solvent followed by the exchange of good solvent with water leads to microphase separation and eventually the final morphology. We hence propose that the lower polymer concentration leads to fewer polymers in each droplet and thus the resultant smaller nanostructure size.

**Fig. 6:**
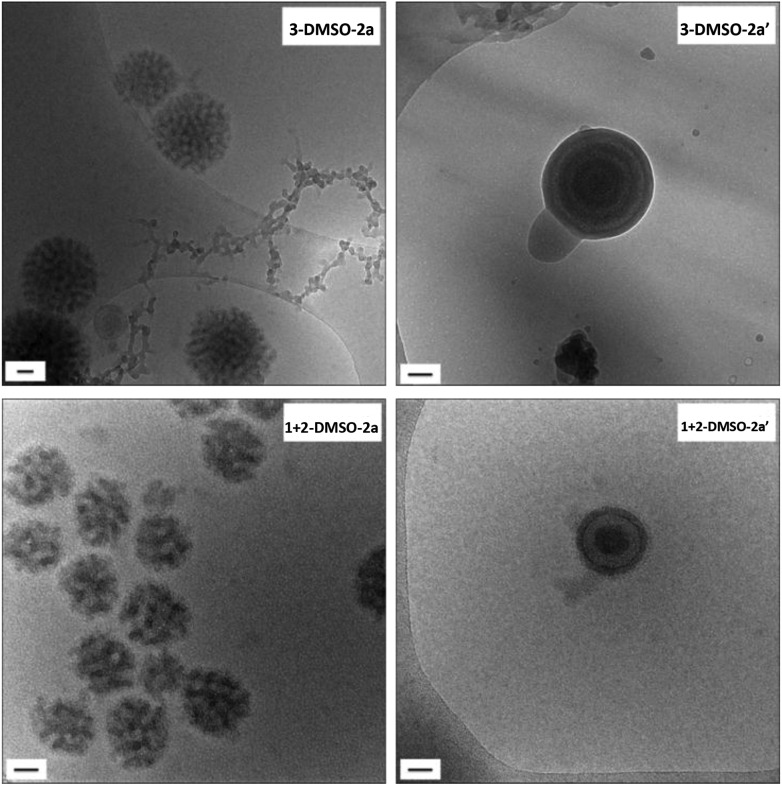
Cryo-TEM images of self-assemblies **3** **-DMSO-2a** and **1** + **2** **-DMSO-2a** and their annealed samples **3** **-DMSO-2a′** and **1** + **2** **-DMSO-2a′**. Scale bar: 50 nm.

The self-assemblies **X-DMSO-2a**, at a concentration of 0.2 mg mL^–1^, were then annealed. The annealing conditions used were the same as for **X-DMSO**, which included 3 heating–cooling cycles with a temperature range from 15 °C to 85 °C (at 1 °C min^–1^). By both cryo-TEM and DLS analysis the annealed samples (**X-DMSO-2a′**) underwent similar morphology transitions to that observed for the samples prepared at a higher concentration, **X-DMSO′** (*i.e.*, a solid-hollow transition was observed for polymer **3**, and a 1 : 1 mixture of **1** and **2** see Fig. 6, S24 and S25†).

## Conclusion

Nucleobase-containing block copolymers have been successfully synthesized by RAFT polymerization, in which POEGMA_70_ was used as the hydrophilic block and nucleobase-containing polymers (A, T and A-*co*-T) comprise the hydrophobic segments. Self-assemblies of A, T, A-*co*-T and A-mix-T copolymers were prepared using the solvent switch method using either DMF or DMSO. Spherical micelles and bicontinuous micelles were obtained respectively when DMF and DMSO were used. We proposed that the reason for this observation was the difference in polymer curvatures resulting from different polymer solubilities in these two common solvents. Furthermore, this study indicates that the morphology of the aggregates formed is determined by the nucleobase compositions rather than the hydrophilic–hydrophobic balance. The presence of adenine–thymine interactions was important for the formation of bicontinuous micelles and key for stabilizing the resultant particles in this study, while individual adenine or thymine polymers exhibited a poor capability of stabilizing particles or producing well-organized structures. Finally, annealing was demonstrated as a promising way to improve the dispersity of the resultant spherical micelles formed in DMF or indeed to induce a bicontinuous micelle to multilamellar vesicle morphology transition. This study highlights the potential to use selective complementary nucleobase interactions to create complex polymeric morphologies which may find application as delivery vehicles or in confined environments for catalysis.

## Conflict of interest

The authors declare no competing financial interest.
