# Supplementary material for: Use of complementary nucleobase-containing synthetic polymers to prepare complex self-assembled morphologies in water
Source: Polym Chem. 2016 Apr 6;7(16):2836–46. doi: 10.1039/c6py00263c (PMC4894073; doi:10.1039/c6py00263c)
Supplement: Supplementary file 1 [file PY-007-C6PY00263C-s001.pdf]

Electronic Supplementary Material (ESI) for Polymer Chemistry.
This journal is © The Royal Society of Chemistry 2016

Supporting Information for

Using complementary nucleobase-containing synthetic polymers to prepare complex self-assembled morphologies in water

Yan Kang,[a] Anaïs Pitto-Barry,[a] Marianne Rolph,[a] Zan Hua,[a] Ian Hands-Portman,[b] Nigel Kirby,[c] Rachel K. O' Reilly[a,]*

[a] *Department of Chemistry, University of Warwick, Library Road., Coventry, CV4 7AL, UK*

[b] *School of Life Sciences, university of Warwick, Gibbet Hill Road, Coventry, CV4 7AL, UK*

[c] *Australian Synchrotron, 800 Blackburn Road, Clayton, Victoria 3168, Australia*

Characterization of monomers 2

Characterization of polymers 3

Self-assembly of polymers **1** - **3** 4

Effect of annealing on self-assembly 12

Effect of different annealing conditions 17

Effect of annealing cycles on resultant morphologies 18

Effect of polymer concentration 20

Characterization of monomers

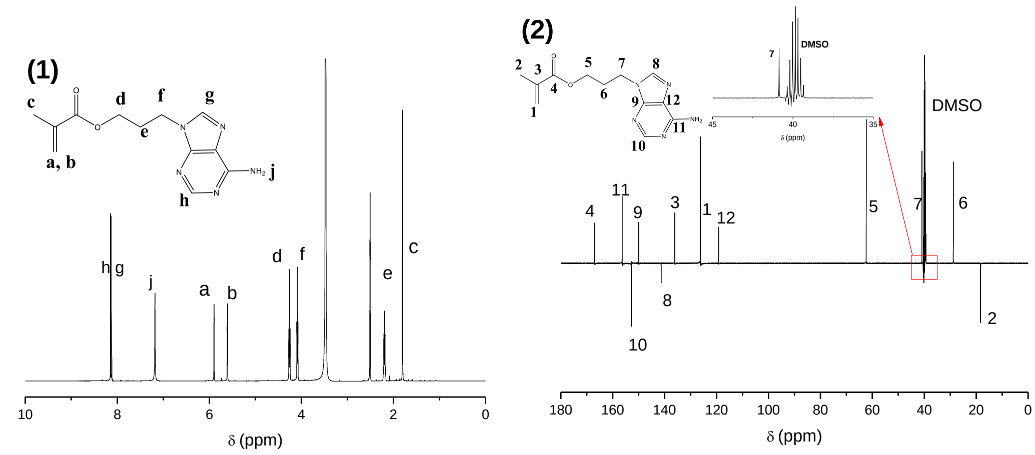

Figure S1. ^{1}H NMR and ^{13}C DEPT NMR spectra of AMA monomer in DMSO-d_6.

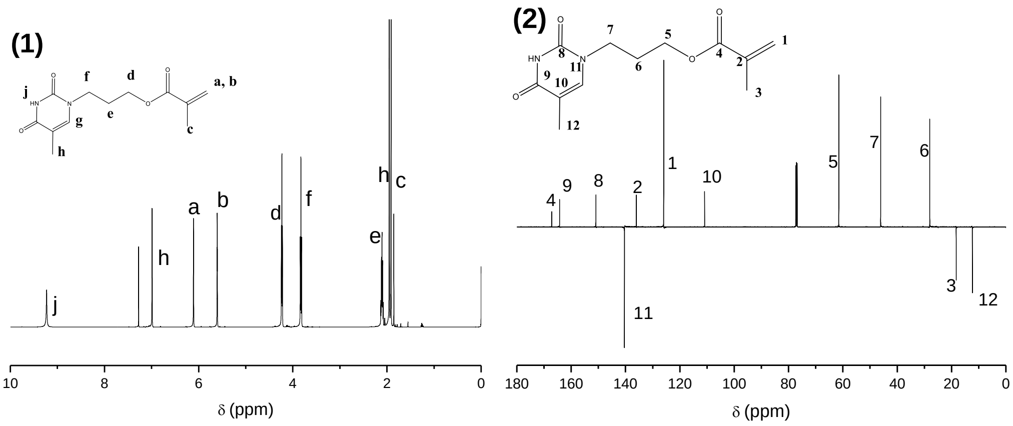

Figure S2. ^{1}H NMR and ^{13}C DEPT NMR spectra of TMA monomer in $CDCl_3$.

Characterization of polymers

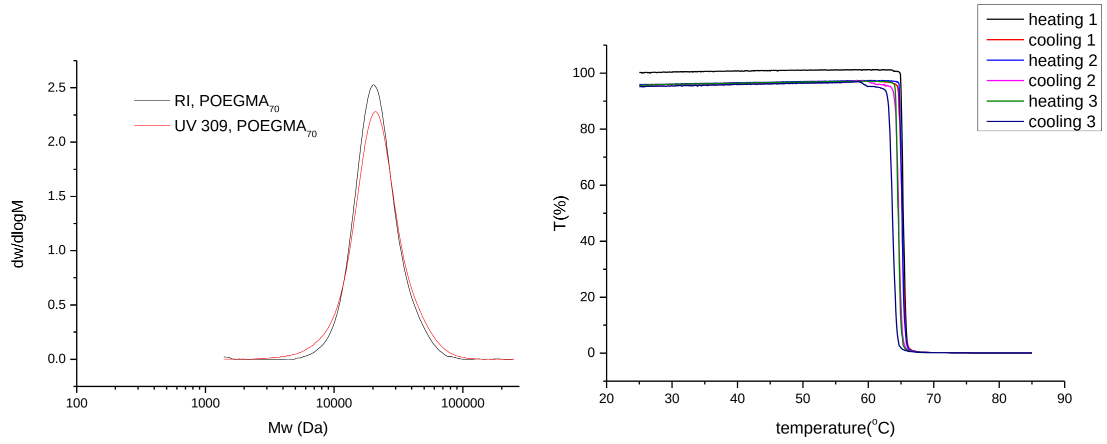

Figure S3. SEC traces of POEGMA$_{70}$ (DMF as eluent, PMMA as standards) and plots of transmittance as a function of temperature (λ = 500 nm, heating/cooling rate 0.5 °C/min) measured for an aqueous solution of POEGMA$_{70}$ (10 mg/mL).

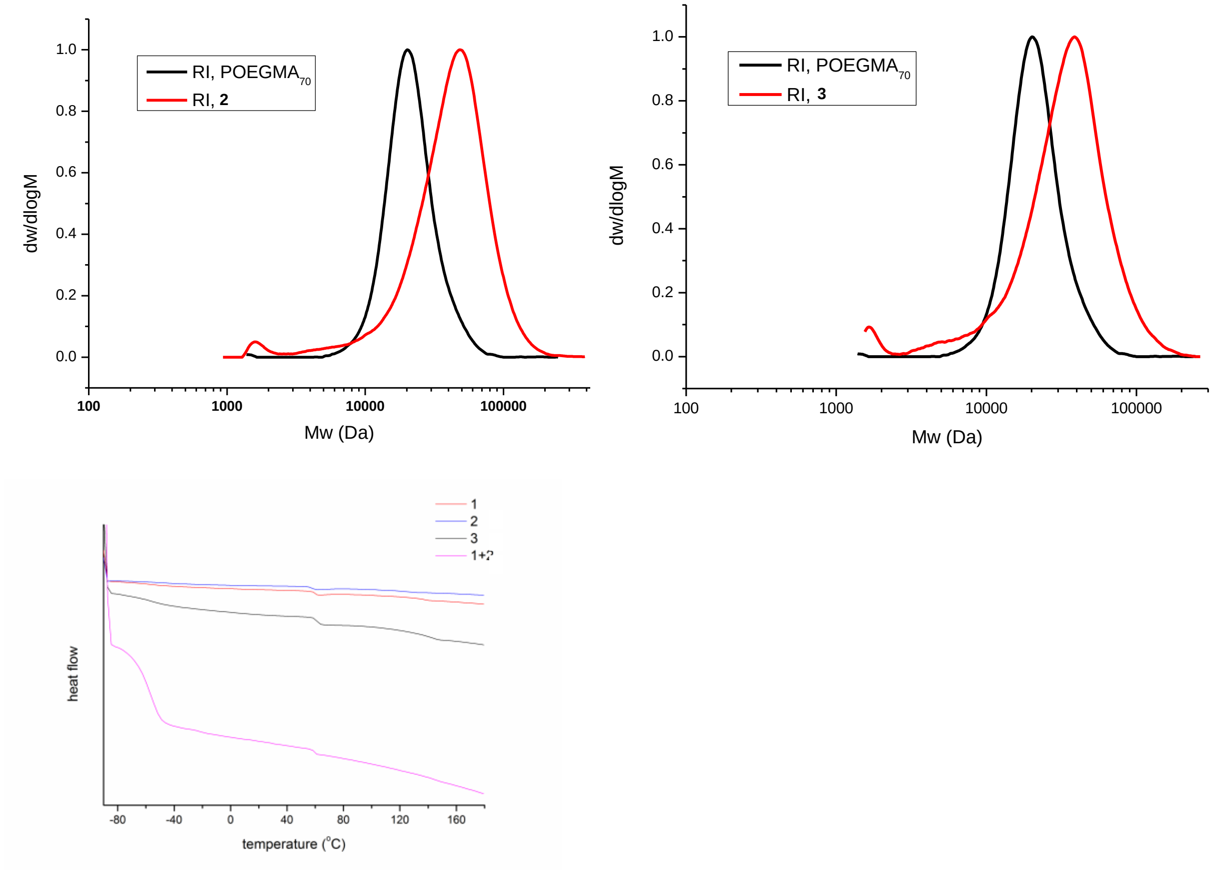

Figure S4. SEC traces and DSC second heating thermograms of POEGMA$_{70}$, polymers **2** and **3** prepared by RAFT polymerization in DMF/DMSO using POEGMA$_{70}$ as the macro-CTA (DMF as eluent, PMMA as standards).

Self-assembly of polymers 1 - 3

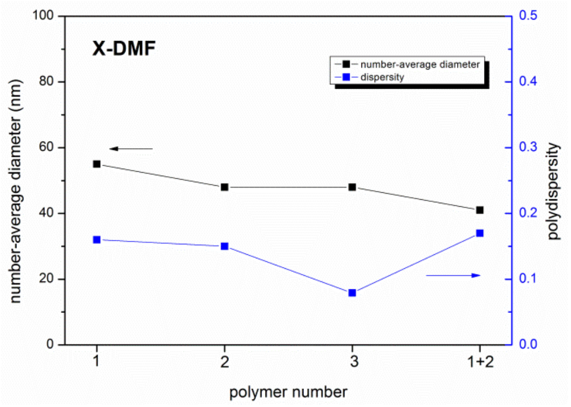

Figure S5. DLS analysis of self-assemblies **1-DMF**, **2-DMF**, **3-DMF** and **1+2-DMF**.

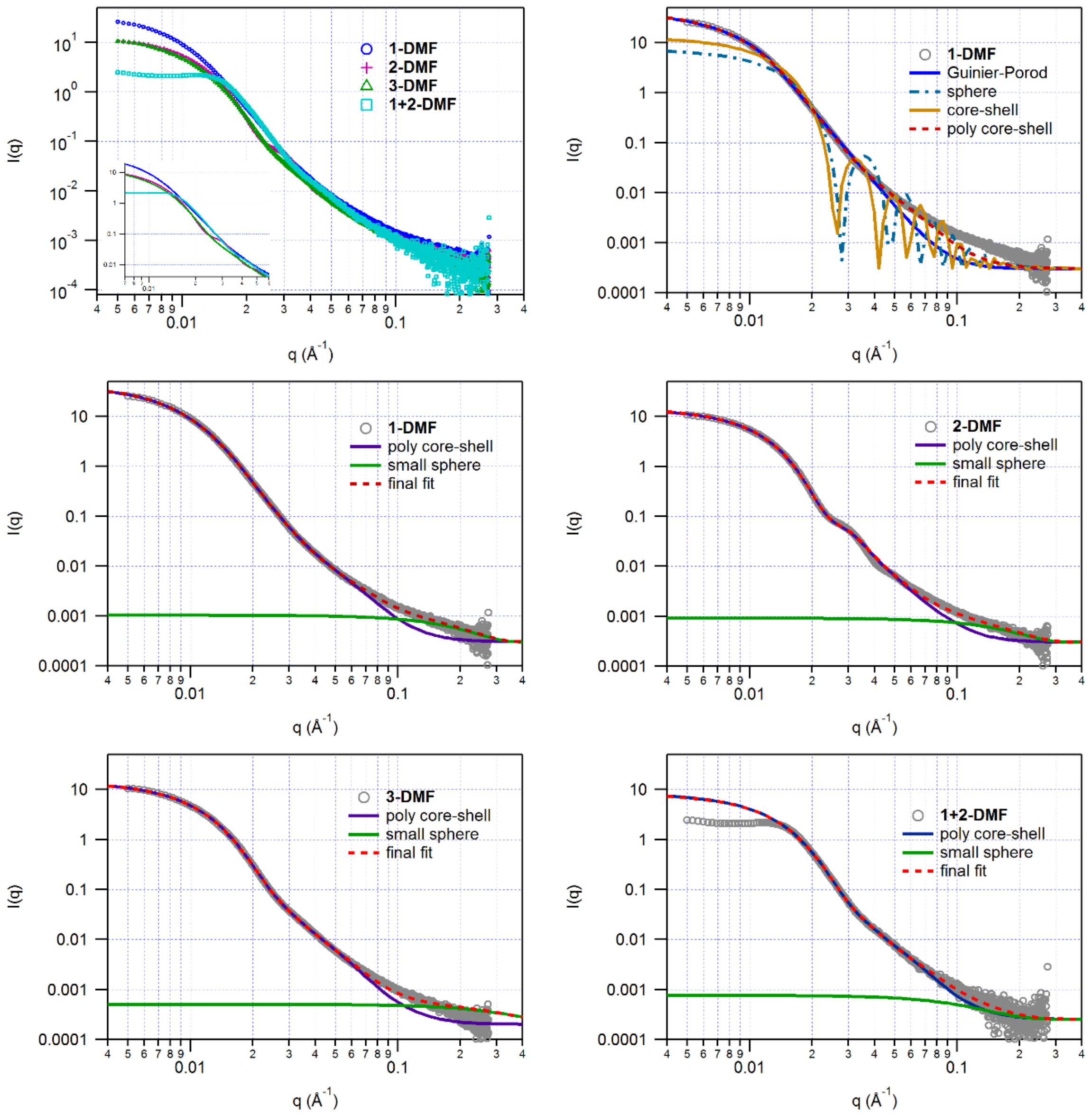

Figure S6. SAXS profiles and fits for **1-DMF**, **2-DMF**, **3-DMF** and **1+2-DMF**. First a Guinier-Porod fit is performed to estimate the R_g and the morphology of the particles. A step-by-step approach is used to gradually increase the complexity of the fits and thus to provide better quality final fits with less errors. A uniform monodisperse spherical model was fitted to give a rough approximation of the radius, then a core-shell structure was introduced (labeled core-shell on graphs), then the complexity was increased by adding some dispersity to the core-shell spherical particles (labeled poly core-shell on graphs). As the poly core shell fit is not satisfactory at high q values, a second population of smaller spheres was added (labeled small sphere on graphs) and a linear summation of these two populations of spheres (labeled final fit on graphs) is created to fit the SAXS profile. Such a pattern for the analysis was used for the 4 samples in DMF.

Table S1. SAXS analysis of samples **1-DMF**, **2-DMF**, **3-DMF** and **1+2-DMF** using a core-shell spherical micelle model with dispersity on the diameter (core-shell with constant core/shell ratio in the NIST package).

	R_{core} (nm)	t_{shell} (nm)	$Đ_M$	Diameter (nm)	SLD_{core} (10^{-6} Å^{-2})[a]	SLD_{shell} (10^{-6} Å^{-2})[a]	R_g (nm)[b]	D_h (nm)[c]	R_g/R_h
**1-DMF**	10.1	6.0	0.37	32.2	6.23	7.03	16.7	55	0.61
**2-DMF**	12.8	4.7	0.20	35.0	7.31	7.44	17.0	48	0.71
**3-DMF**	8.0	8.3	0.27	32.6	7.07	7.55	15.4	48	0.64
**1+2-DMF**	6.9	6.7	0.25	27.2	7.00	7.47	13.7	41	0.67

[a]SLD: scattering length density, as estimated by the final fit (linear summation of the big and small sphere models), the SLD value of the core for **1+2-DMF** is greater than the one for **1-DMF** and smaller than the one for **2-DMF**, which suggests a different packing of the chains in the core of the micelle. [b] Value determined by fitting the SAXS profile with the Guinier-Porod model available in the NIST package. [c] Value determined by DLS.

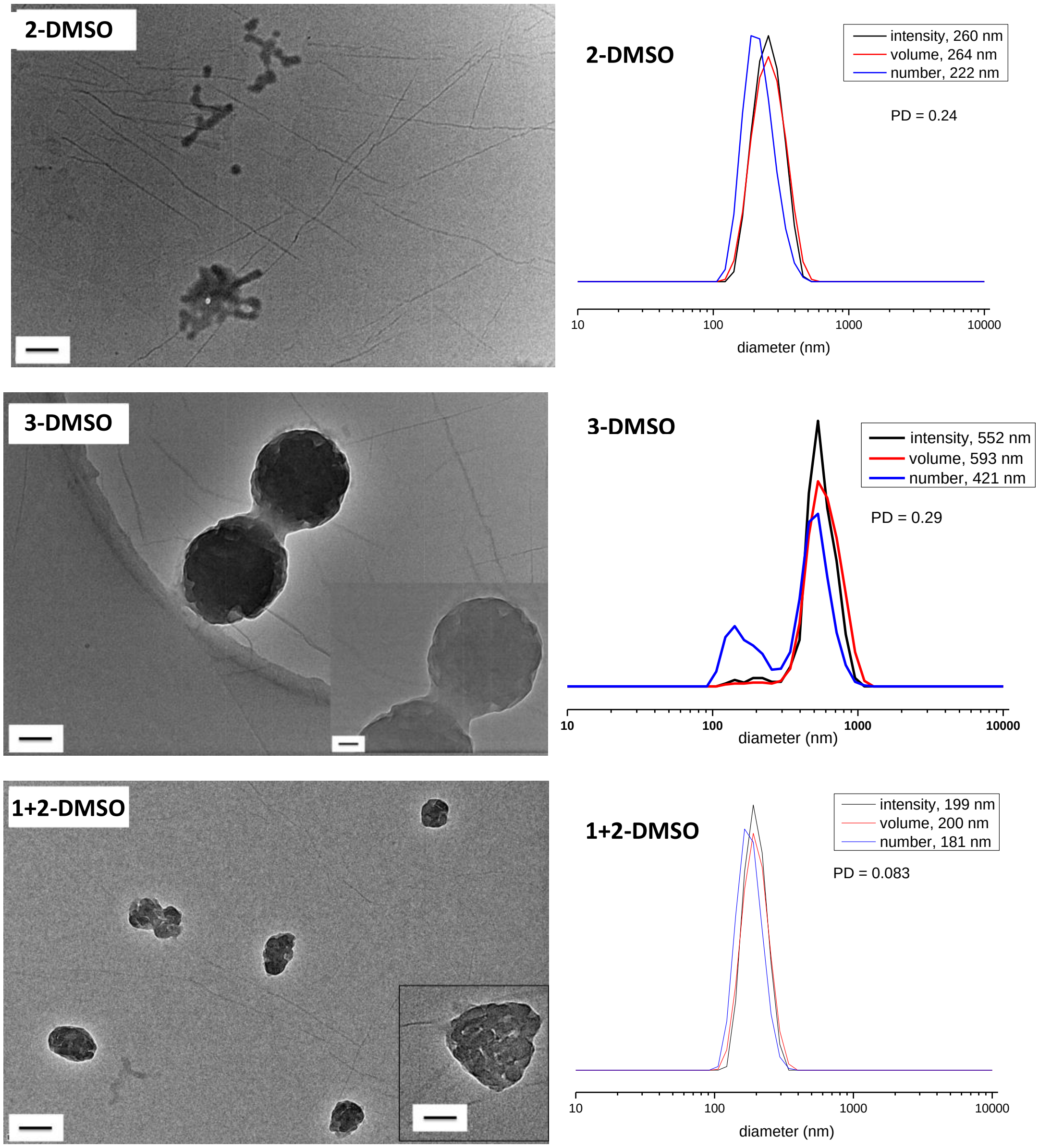

Figure S7. Representative dry-state unstained TEM images on GO-coated grids and DLS analysis of self-assemblies prepared from polymers **2**, **3**, a 1:1 mixture of **1** and **2** by a solvent-switch method using DMSO as the common solvent: **2-DMSO**, **3-DMSO**, and **1+2-DMSO**. Scale bar: 100 nm (inset 50 nm).

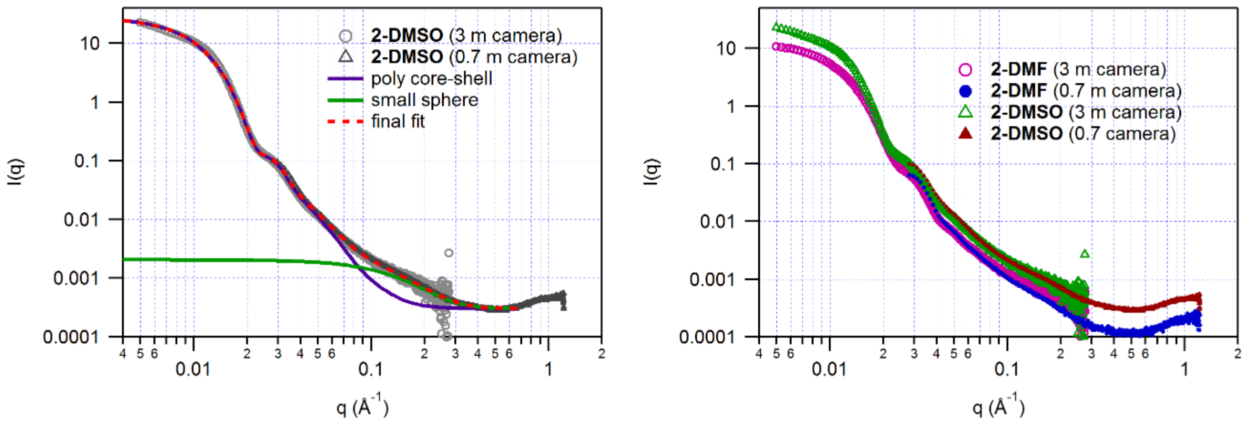

Figure S8. SAXS profiles and fits for **2-DMSO**, and comparison of the SAXS profiles for **2** in DMF and DMSO at the different sample-to-detector lengths (3 and 0.7 m). A process similar to the one used for the DMF samples was used for **2-DMSO** to obtain a final fit (linear summation of a main polydisperse core-shell spherical micelle population and a secondary population of smaller spherical micelles). Profiles for **3-DMSO** and **1+2-DMSO** exhibit a different pattern with the presence of at least one peak that accounts for a periodic pattern within the structure of the nanoparticles. Particles are too big (from TEM and DLS analysis) to be fully seen using the SAXS set-up, explaining why the plateau at low q values is not reached. Fits were done on the 3 meter SAXS profile (light grey circle). It is interesting to notice that the fits correlate perfectly with the high q values of the 0.7 meter SAXS profile too (dark grey triangle), this confirms the quality of the modelling. A visual inspection of the profiles in the different solvents confirms that structures are slightly larger in DMSO than in DMF, as the curvature of the oscillations is at lower q values for **2-DMSO** than for **2-DMF**.

Table S2. SAXS analysis of samples **2-DMF** and **2-DMSO** using a core-shell spherical micelle model with dispersity on the diameter (core-shell with constant core/shell ratio in the NIST package).

	R_{core} (nm)	t_{shell} (nm)	$Đ_M$	Diameter (nm)	SLD_{core} (10^{-6} Å^{-2})[a]	SLD_{shell} (10^{-6} Å^{-2})[a]	R_g (nm)[b]	D_h (nm)[c]	R_g/R_h
**2-DMF**	12.8	4.7	0.20	35.0	7.31	7.44	17.0	48	0.71
**2-DMSO**	9.7	9.4	0.16	40.2	6.40	6.76	18.2	222	-

[a]SLD: scattering length density, as estimated by the final fit (linear summation of the big and small sphere models). [b] Value determined by fitting the SAXS profile with the Guinier-Porod model available in the NIST package. [c] Value determined by DLS. It is interesting that big assemblies are observed by DLS while both TEM and SAXS analysis confirm the presence of discrete spherical particles of 40 nm in diameter. TEM images present some aggregation of the spherical micelles that leads to the formation of bigger clusters, as seen by DLS.

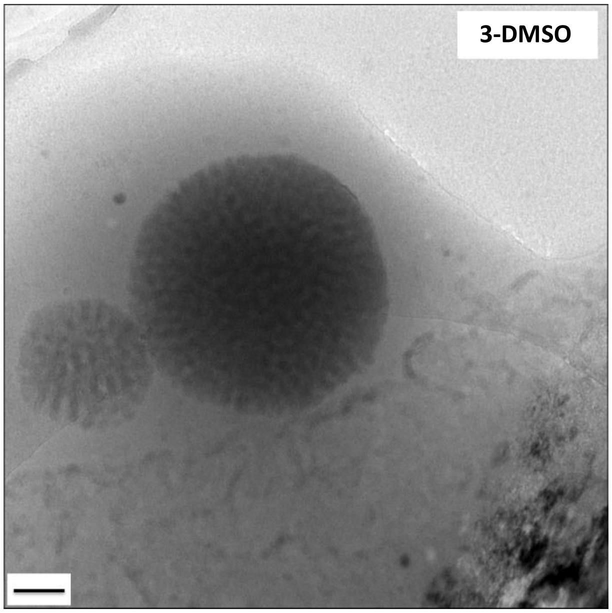

Figure S9. Cryo-TEM image of self-assemblies **3-DMSO** at a concentration of 0.2 mg/mL (scale bar: 100 nm).

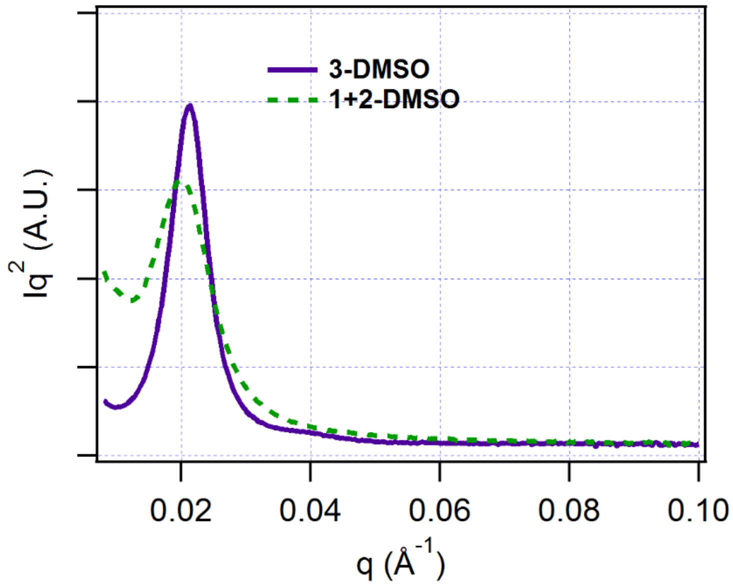

Figure S10. Kratky plots of self-assemblies **3-DMSO** and **1+2-DMSO**. Both the bell-like shape and the horizontal asymptote at high *q* values are characteristic of compact spherical objects.

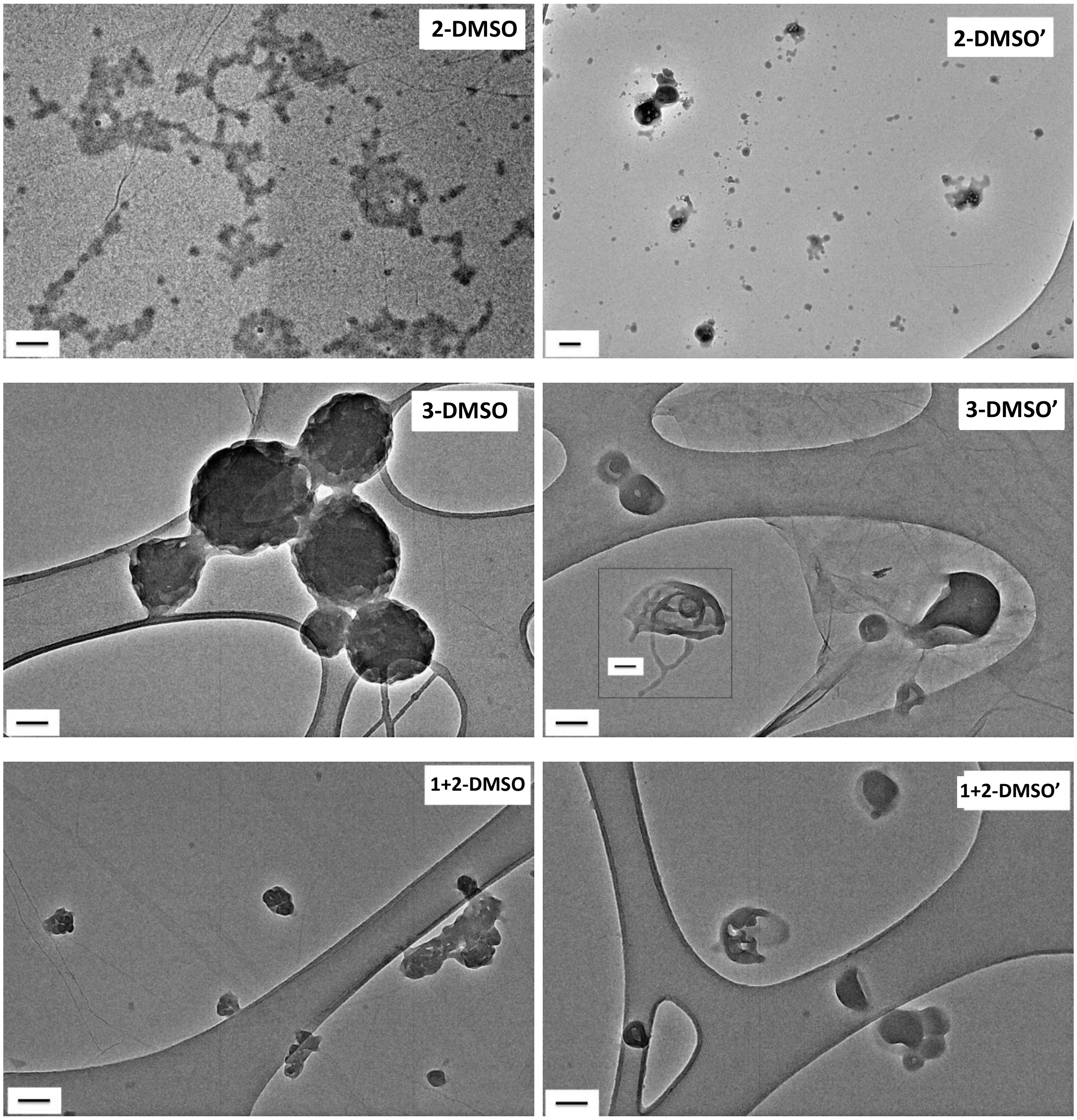

Figure S11. Representative unstained dry-state TEM images on GO grids of self-assemblies after 6 months **2-DMSO**, **3-DMSO**, and **1+2-DMSO** and their corresponding annealed self-assemblies **2-DMSO’**, **3-DMSO’**, and **1+2-DMSO’** (annealing conditions: 15 °C – 85 °C and then 85 °C – 15 °C with a rate of 1 °C/min for 3 cycles). Scale bar: 100 nm (inset 50 nm).

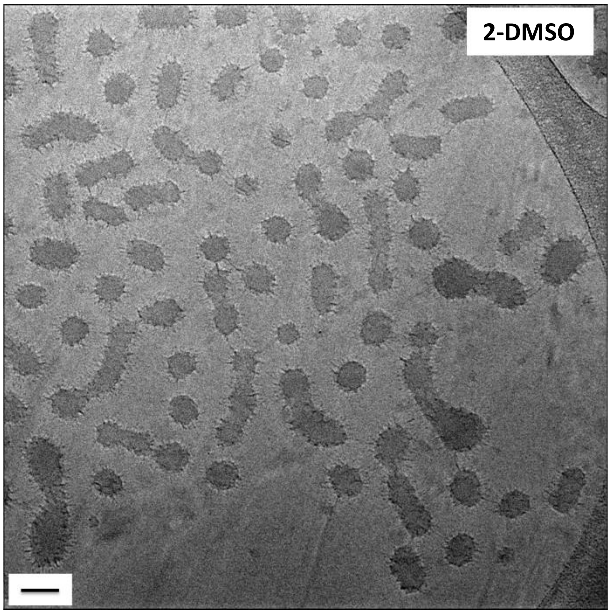

Figure S12. Representative cryo-TEM of self-assembly **2-DMSO** after 6 months (scale bar: 50 nm).

Effect of annealing on self-assembly

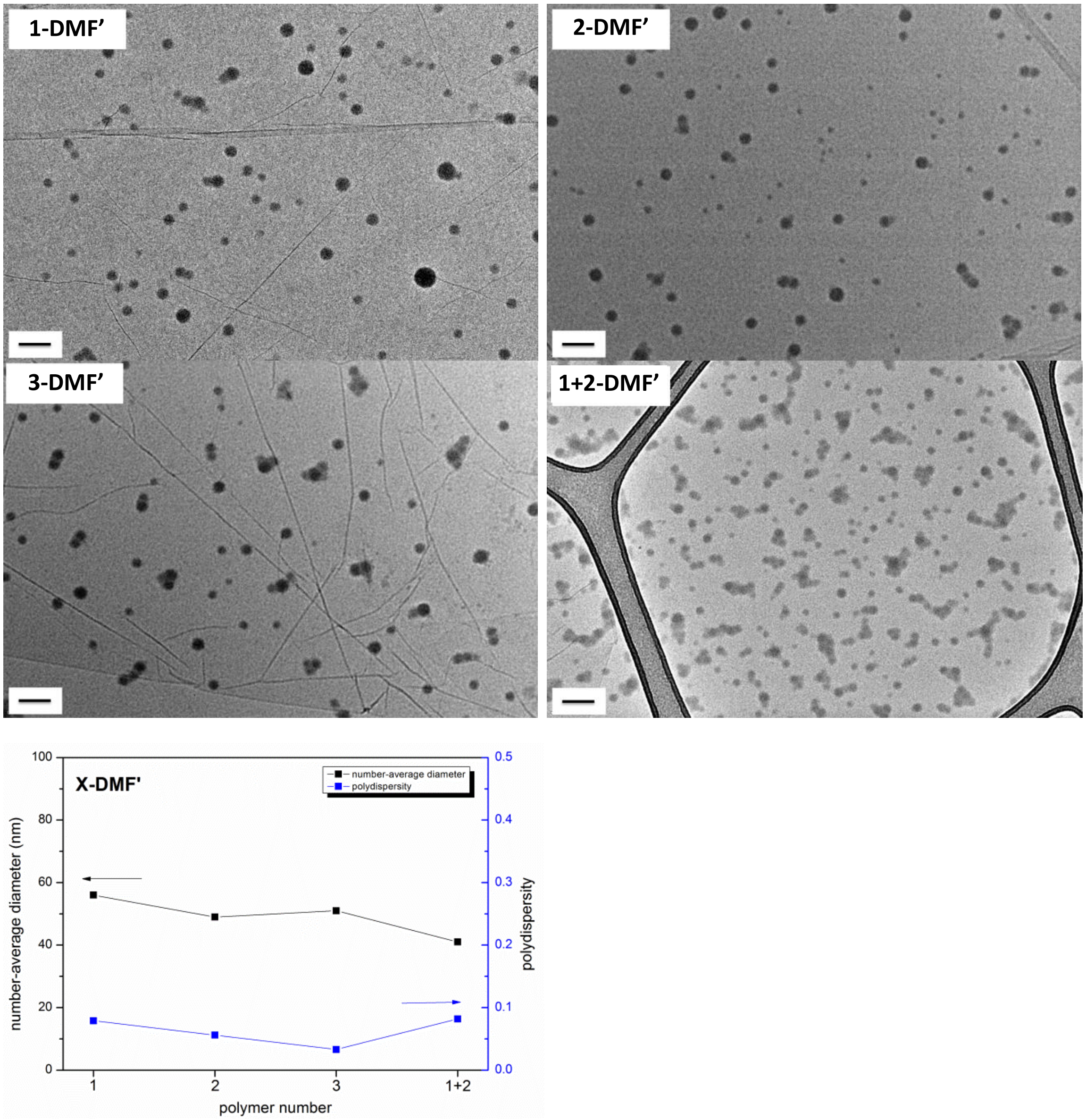

Figure S13. Representative unstained dry-state TEM images on GO grids and DLS analysis of self-assemblies **1-DMF’**, **2-DMF’**, **3-DMF’** and **1+2-DMF’** (annealing conditions: 15 – 85 °C and then 85 – 15 °C with a rate of 1 °C/min for 3 cycles). Scale bar: 100 nm.

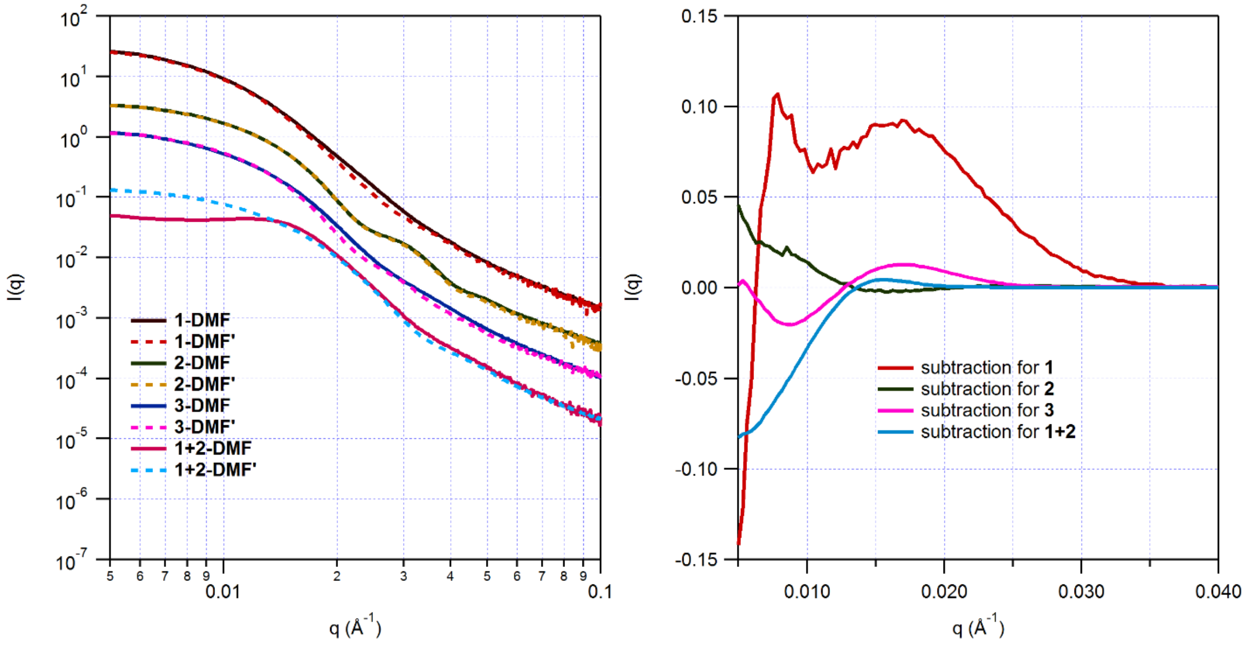

Figure S14. SAXS profiles of the assemblies in DMF before and after annealing. All profiles have been shifted vertically to allow for easy comparison of the profiles for the same polymer. The right graph is the subtraction of the assembly from the annealed assembly, emphasizing the differences between the profiles, especially in the q range 0.015-0.060 $Å^{-1}$. The assemblies after annealing exhibit a lower dispersity, as determined from the SAXS profiles as the harmonics are more visible (for example **3-DMF’** *vs* **3-DMF**).

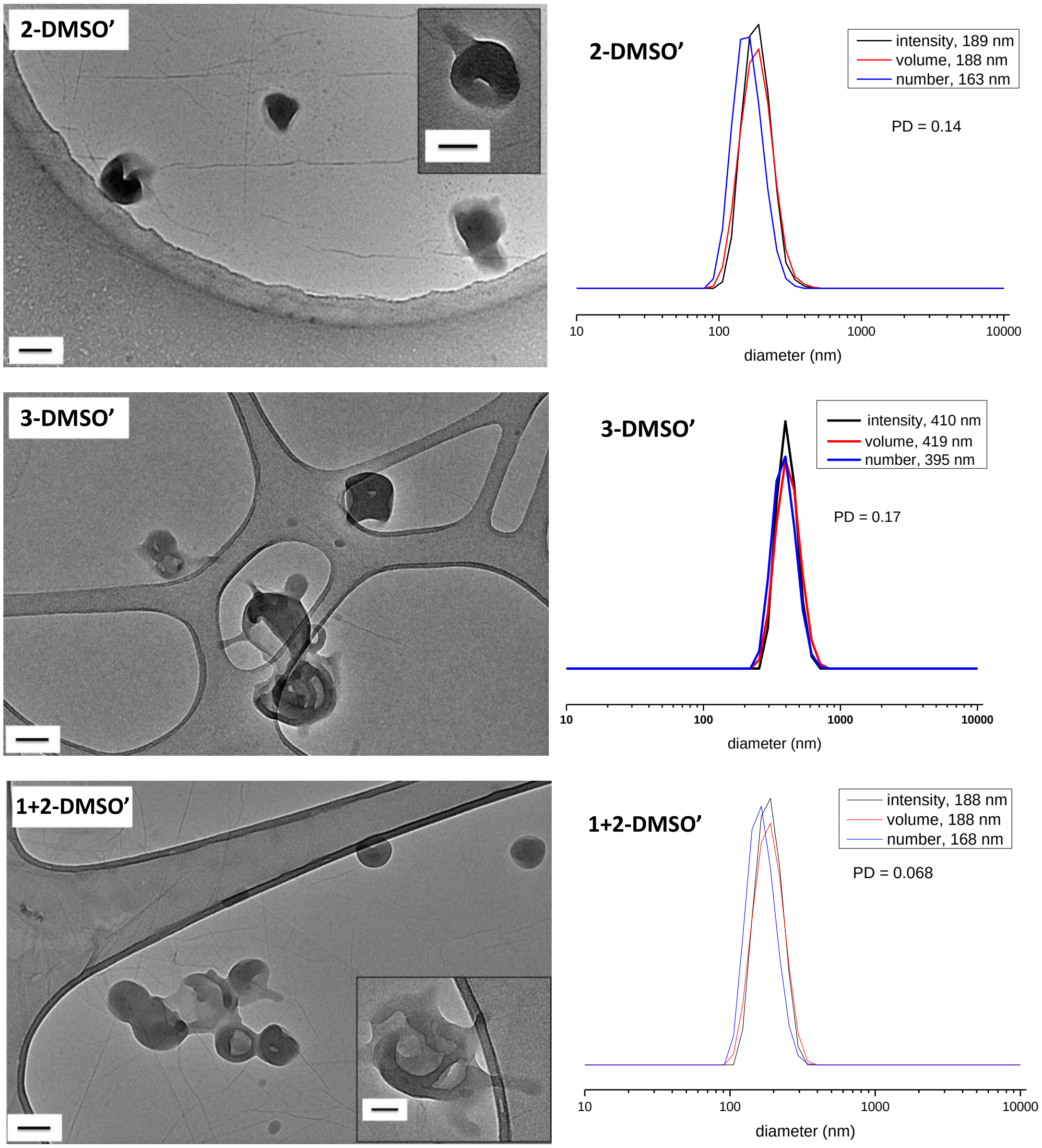

Figure S15. Representative unstained dry-state TEM images on GO grids and DLS analysis of self-assemblies **2-DMSO'**, **3-DMSO'** and **1+2-DMSO'** (annealing conditions: 15 – 85°C and then 85 – 15 °C with a rate of 1 °C/min for 3 cycles). Scale bar: 100 nm.

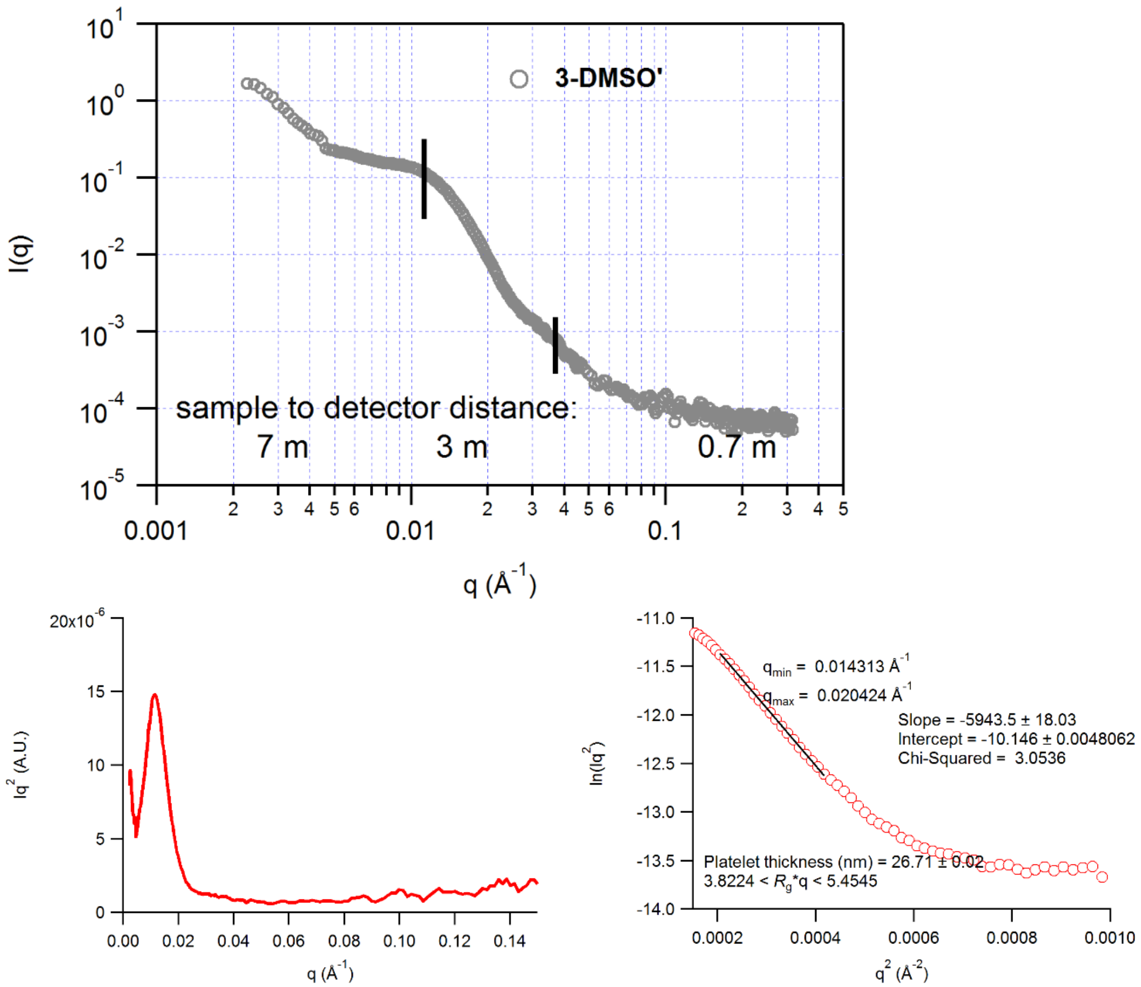

Figure S16. SAXS profile of the assembly **3-DMSO'**. The profile is the merged curve of the three individual profiles at the three sample-to-detector distances. The bottom left graph is a Krakty plot which shows the spherical morphology in solution as there is a bell-like shape at low *q* values and an horizontal asymptote at high *q* values. The bottom right graph is a Kratky-Porod plot and is used to determine the R_g (from the slope, equals to $-R_g^2$) and the thickness of the vesicle's shell (vesicles are assumed to be sheet-like objects).

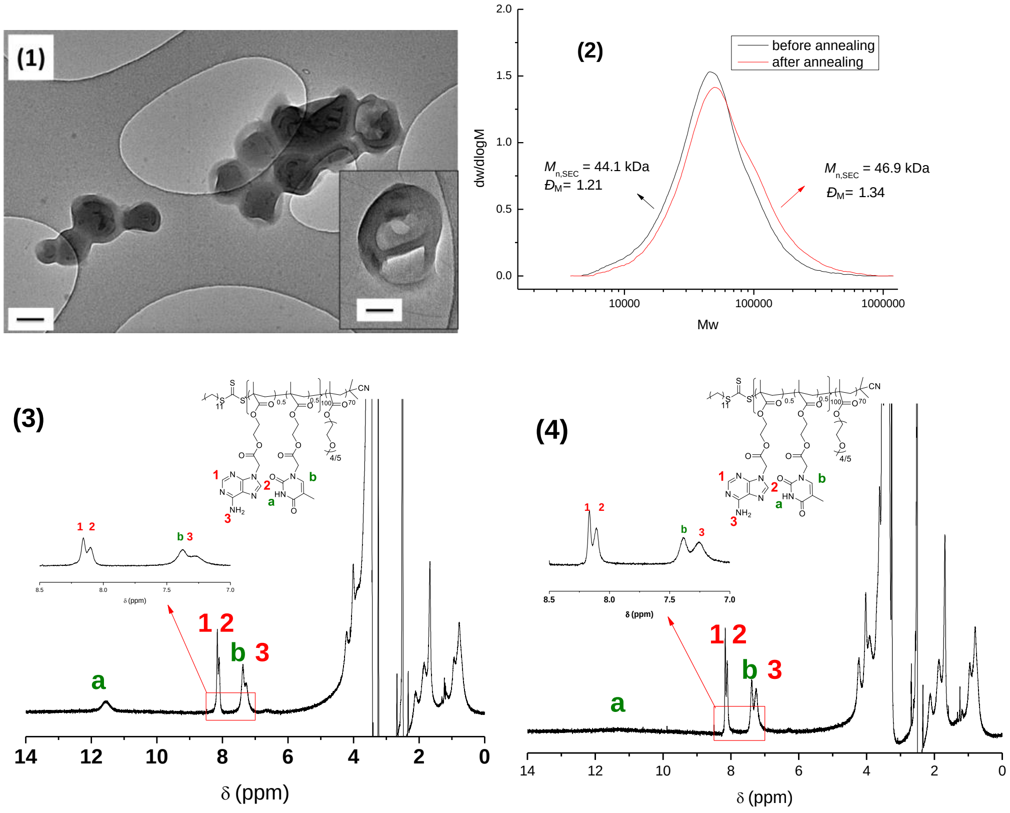

Figure S17. Representative unstained dry-state TEM image on GO grids of **3-DMSO’** with a concentration of 1 mg/mL (1); SEC traces of polymer **3** harvested from solution **3-DMSO** and **3-DMSO’** (2); ^{1}H NMR spectra of polymer **3** harvested from solution: **3-DMSO** (3); **3-DMSO’** (4).

Effect of different annealing conditions

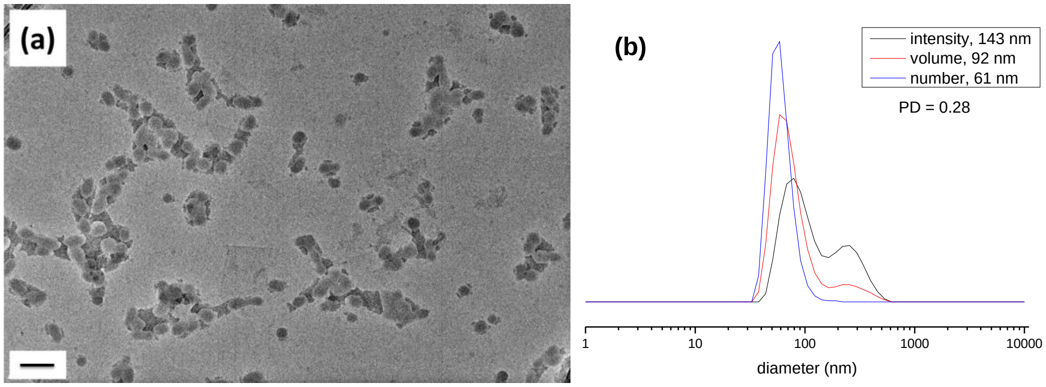

Figure S18. Representative unstained dry-state TEM images on GO grids and DLS analysis of **3-DMSO** heated at 85 °C for 45 min and then cooled down in an oil bath naturally. Scale bar: 100 nm.

Effect of annealing cycles on resultant morphologies

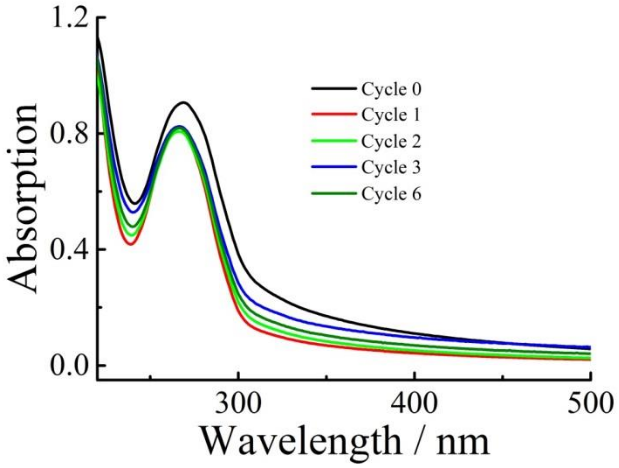

Figure S19 Evolution of UV-vis signal for **3-DMSO** upon 6 annealing cycles.

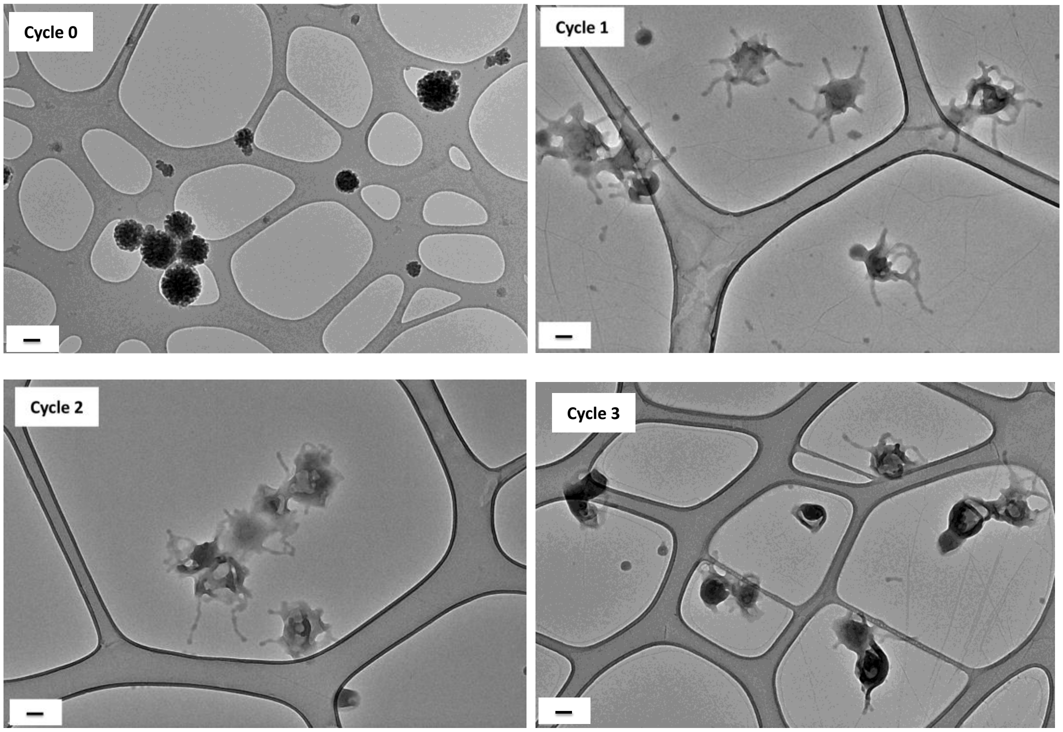

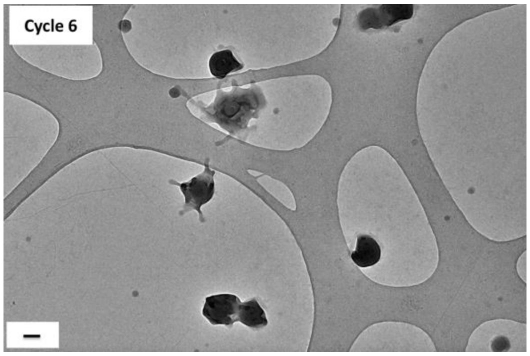

Figure S20. Evolution of self-assembly from **3-DMSO** to **3-DMSO’** with annealing cycles characterized by unstained dry-state TEM images on GO grids (scale bar = 100 nm).

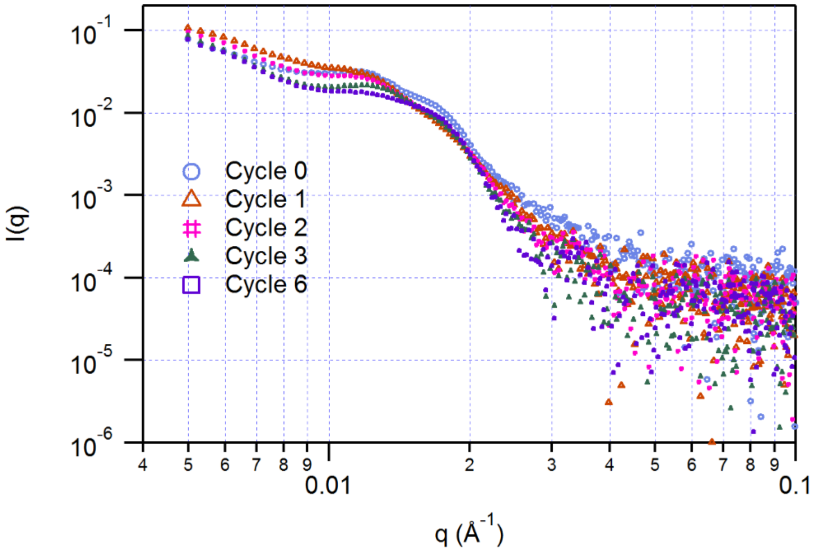

Figure S21. Evolution of the SAXS profiles of self-assembly from **3-DMSO** to **3-DMSO’** with 6 annealing cycles.

Effect of polymer concentration

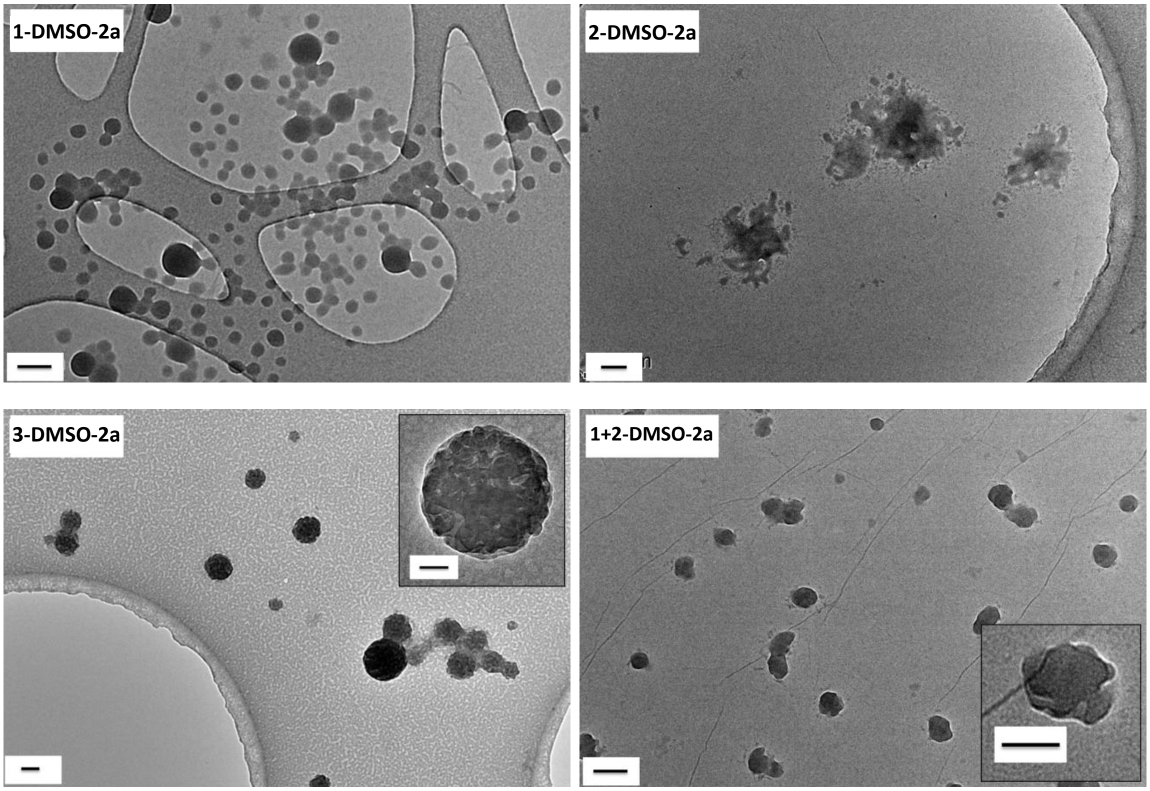

Figure S22. Representative TEM (unstained dry-state TEM images on GO grids) analysis of self-assemblies prepared from polymers **1**, **2**, **3**, and a 1:1 mixture of **1** and **2** with an initial concentration of 2 mg/mL by solvent-switch method using DMSO as the common solvent: **1-DMSO-2a**, **2-DMSO-2a**, **3-DMSO-2a**, and **1+2-DMSO-2a**. Scale bar: 100 nm (inset 50 nm).

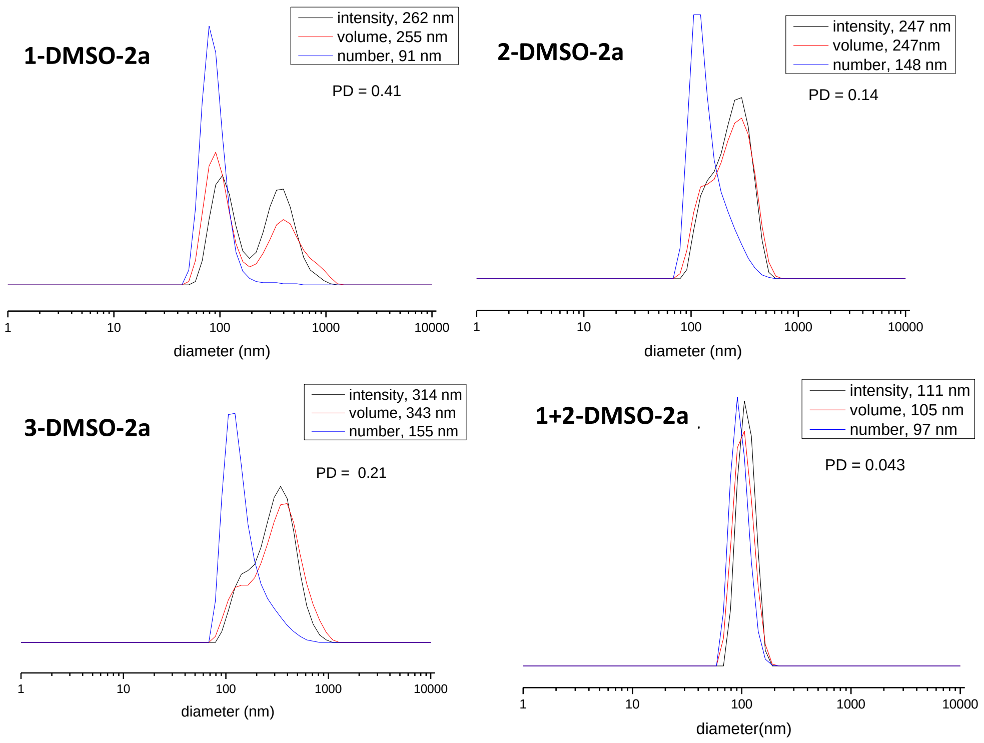

Figure S23. DLS analysis of self-assemblies prepared from polymers **1**, **2**, **3**, and a 1:1 mixture of **1** and **2** with an initial concentration of 2 mg/mL by solvent-switch method using DMSO as the common solvent: **1-DMSO-2a**, **2-DMSO-2a**, **3-DMSO-2a**, and **1+2-DMSO-2a**.

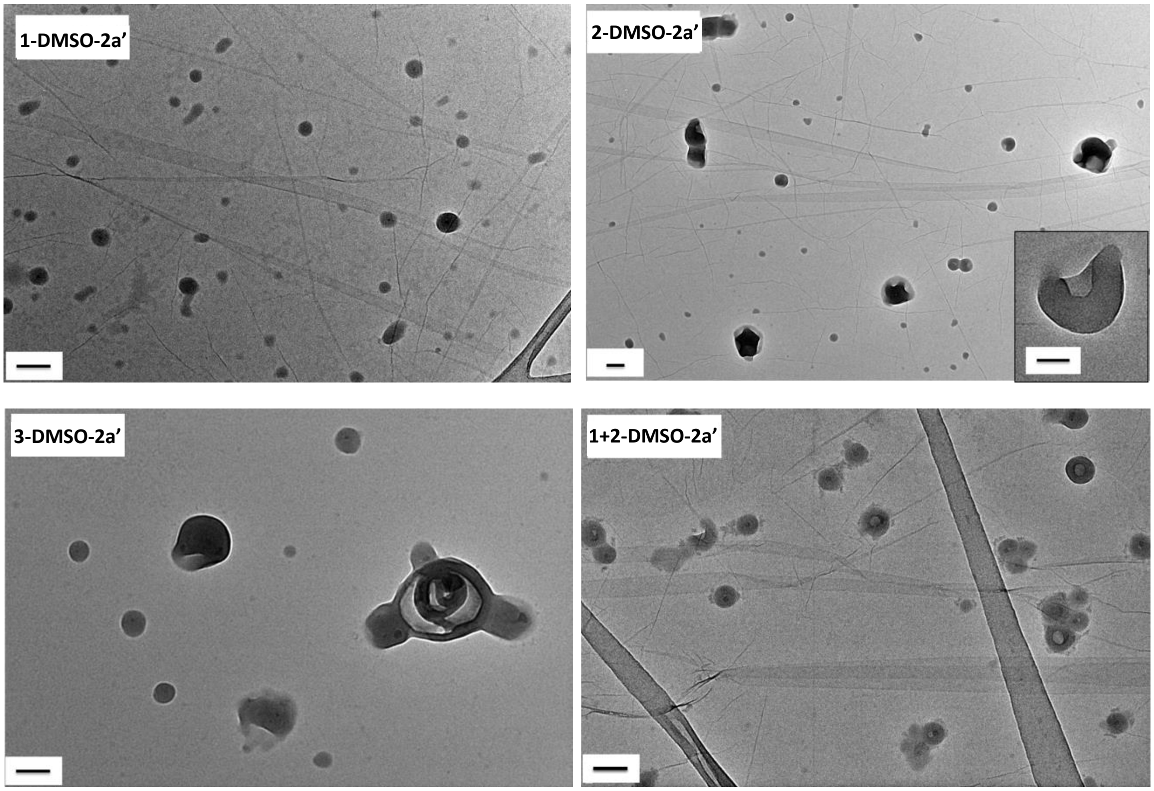

Figure S24. Representative TEM analysis (unstained dry-state TEM images on GO grids) of self-assemblies **1-DMSO-2a'**, **2-DMSO-2a'**, **3-DMSO-2a'**, and **1+2-DMSO-2a'** prepared by annealing **1-DMSO-2a**, **2-DMSO-2a**, **3-DMSO-2a**, and **1+2-DMSO-2a**, (annealing conditions: 15 °C – 85 °C and then 85 °C – 15 °C with a rate of 1 °C/min for 3 cycles). Scale bar: 100 nm (inset 50 nm).

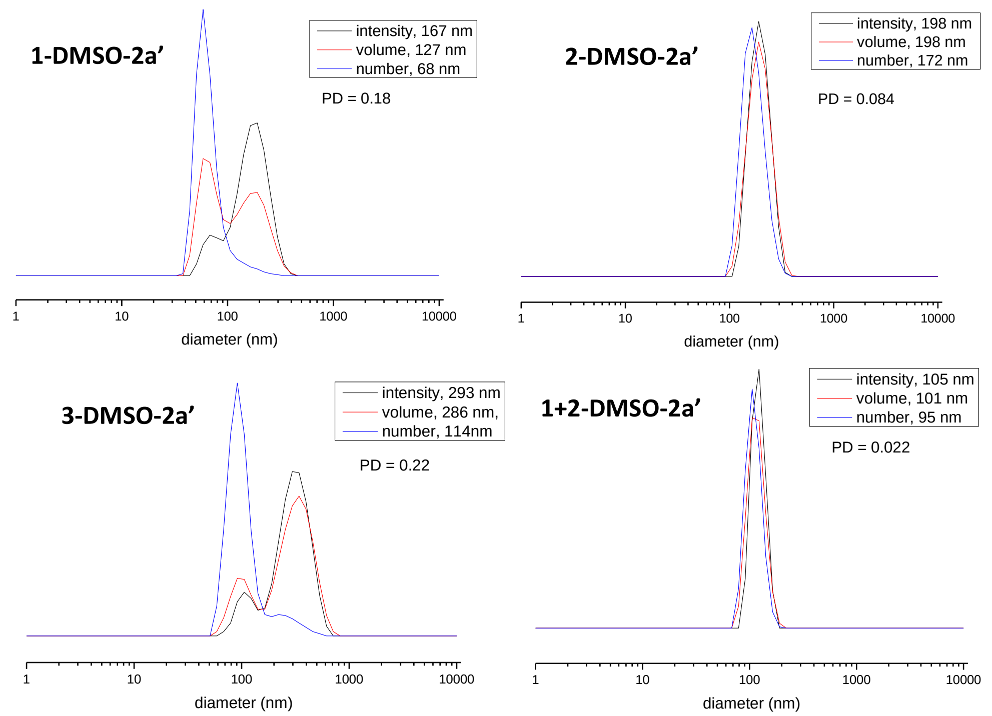

Figure S25. DLS analysis of self-assemblies **1-DMSO-2a’**, **2-DMSO-2a’**, **3-DMSO-2a’**, and **1+2-DMSO-2a’** prepared by annealing **1-DMSO-2a**, **2-DMSO-2a**, **3-DMSO-2a**, and **1+2-DMSO-2a**, (annealing conditions: 15 °C – 85 °C and then 85 °C – 15 °C with a rate of 1 °C/min for 3 cycles).
